# Narrow Therapeutic Index Drugs: Regulatory Frameworks, Bioequivalence, and Model-Informed Precision Dosing

**DOI:** 10.3390/pharmaceutics18091190

**Published:** 2026-09-20

**Authors:** Mengmeng Liu, Zhe Wang, Yongbo Chen, Yuqi Jia, Lei Xu, Haojie Xu, Jueyu Li, Libo Zhao

**Affiliations:** Department of Pharmacy, Peking University Third Hospital, Beijing 100191, China; 2511210484@bjmu.edu.cn (M.L.); zhewang1224@163.com (Z.W.); 18868284537@163.com (Y.C.); 18221009311@163.com (Y.J.); xuleicpu6657@163.com (L.X.); x17852205820@163.com (H.X.); 19818986136@163.com (J.L.)

**Keywords:** narrow therapeutic index drugs, bioequivalence, generic substitution, model-informed precision dosing, therapeutic drug monitoring, population pharmacokinetics, physiologically based pharmacokinetics, machine learning

## Abstract

**Background/Objectives**: Narrow therapeutic index (NTI) drugs are characterized by a small margin between effective and toxic exposure. This review aimed to summarize international definitions and regulatory frameworks for NTI drugs, compare bioequivalence requirements for generic NTI products, and evaluate the role of model-informed approaches in precision dosing and lifecycle risk management. **Methods**: A structured literature search was conducted in PubMed, MEDLINE, Embase, Web of Science, Scopus, and the China National Knowledge Infrastructure from database inception to 30 June 2026. Official documents from major regulatory authorities were also reviewed. Evidence relating to NTI drug classification, bioequivalence, interchangeability, therapeutic drug monitoring, population pharmacokinetics, physiologically based pharmacokinetics, machine learning, and hybrid modeling was included. **Results**: Definitions, drug lists, study designs, and bioequivalence criteria varied substantially across jurisdictions. Several authorities use narrowed acceptance ranges, whereas China and the United States apply fully replicated crossover designs with reference-scaled average bioequivalence, average bioequivalence constraints, and within-subject variability comparisons for selected NTI drugs. Population pharmacokinetic and Bayesian approaches remain the most mature tools for therapeutic drug monitoring and individualized dosing. Physiologically based models support drug–drug interaction prediction, special-population extrapolation, virtual bioequivalence, and clinically relevant formulation specifications. Machine-learning and hybrid models improve exposure, dose, and toxicity-risk prediction, but most evidence remains retrospective and externally limited. **Conclusions**: NTI drug regulation and clinical management remain heterogeneous. Greater international harmonization, standardized model validation, prospective clinical evaluation, and integration of regulatory, formulation, and patient-level data are needed to support safer substitution and more reliable precision dosing.

## 1. Introduction

Narrow therapeutic index (NTI) drugs are agents for which small changes in dose or blood concentration may result in serious treatment failure or life-threatening, persistent, or significantly disabling adverse effects [1,2]. Their clinical risks arise not only from the narrow separation between effective and toxic doses or concentrations, but also from steep exposure–response relationships, substantial interindividual pharmacokinetic and pharmacodynamic variability, and limited flexibility for dose adjustment [3]. NTI drugs are used across a broad range of therapeutic areas, including anticoagulation, cardiovascular disease, neurological disorders, respiratory disease, and immunosuppression; representative examples include warfarin, digoxin, phenytoin, lithium, theophylline, cyclosporine, and tacrolimus [4]. Accordingly, maintaining therapeutic efficacy while minimizing the risks of underexposure and overexposure remains a common challenge in the development, regulatory evaluation, and clinical use of these drugs.

Generic substitution is an important strategy for reducing drug costs and improving access to treatment; however, the pharmacological characteristics of NTI drugs make the assessment of interchangeability more complex than that for conventional drugs [5]. For these agents, even small differences in systemic exposure between formulations may raise concerns about reduced efficacy or increased toxicity, while sequential switching among different generic products may introduce a risk of so-called “biocreep” [5,6]. “Biocreep” arises when multiple generic products each meet bioequivalence criteria against the same reference product, but their exposure estimates occupy different positions within the acceptance range. Consequently, switching between these generic products may still produce substantial differences in drug exposure. For example, postapproval studies indicated that Accord Healthcare’s generic tacrolimus oral capsules might produce a higher peak blood concentration than the reference product, Prograf. Given the associated toxicity risk, the FDA changed the products’ therapeutic equivalence rating from AB to BX [5]. Potential biocreep therefore complicates regulatory oversight of narrow therapeutic index (NTI) drugs. Evaluations should extend beyond generic–reference bioequivalence to consider generic-to-generic interchangeability, complementary exposure metrics, and continued postapproval reassessment.

The approval of generic drugs typically relies on bioequivalence studies that bridge to the established efficacy and safety evidence for the reference product. Regulatory assessment must therefore establish that formulation differences do not produce clinically meaningful changes in drug exposure. For narrow therapeutic index (NTI) drugs, the margin between therapeutic and toxic exposure is small. Even modest changes in exposure may therefore compromise efficacy or safety, providing a scientific rationale for stricter or product-specific bioequivalence criteria. Accordingly, some regulatory authorities apply narrower bioequivalence acceptance ranges, whereas others use fully replicated crossover designs. These designs estimate within-subject variability for the reference product and compare the test and reference products in terms of both mean exposure and within-subject variability [7,8]. Nevertheless, substantial differences remain across countries and regions in the terminology, classification criteria, drug lists, study designs, and BE acceptance standards applied to NTI drugs. These inconsistencies limit the cross-regional comparability of study findings and complicate global drug development and clinical substitution decisions [9]. A recent analysis of generic drug applications submitted to the U.S. Food and Drug Administration (FDA) further showed that BE assessment frameworks for different NTI drugs vary in stringency, study pass rates, and statistical error control, indicating a continued need to balance scientific rigor, practical feasibility, and international regulatory harmonization [10]. Current regulatory frameworks lack a consistent cross-jurisdictional approach to narrow therapeutic index (NTI) drug designation, pharmacokinetic endpoint selection, study design, and bioequivalence acceptance limits. These regulatory decisions are not consistently aligned with drug-specific clinical risks.

The choice of parameters for bioequivalence assessment should reflect the exposure characteristics that formulation differences may alter and the clinical relevance of those changes. AUC_0–t_ quantifies cumulative systemic exposure from dosing to the last quantifiable concentration, whereas C_max_ captures peak exposure and is influenced by the absorption rate. These measures therefore serve as the primary endpoints in conventional pharmacokinetic bioequivalence assessments. AUMC is the area under the first moment curve of the concentration–time profile, whereas MRT is generally calculated as the ratio of AUMC to AUC. After oral administration, MRT reflects both drug absorption and subsequent disposition. Although AUMC and MRT characterize the temporal profile of systemic exposure, neither directly substitutes for measures of total or peak exposure. Their estimates are also particularly sensitive to inadequate terminal sampling and the proportion of the curve requiring extrapolation. They are therefore generally treated as supplementary pharmacokinetic parameters rather than primary endpoints for routine bioequivalence decisions. When early exposure, onset of action, or steady-state trough concentration is clinically relevant, partial AUC, t_max_, C_τ_, or other appropriate measures may be included. The choice should reflect the drug, formulation, and specific clinical question. For narrow therapeutic index (NTI) drugs, endpoint selection and the definition of acceptance limits are related but distinct decisions. Endpoint selection should identify the exposure dimension requiring comparison on the basis of the exposure–response relationship. Acceptance limits should instead reflect the clinical consequences of small exposure differences, within-subject variability, and applicable product-specific guidance. Suitable endpoints and acceptance criteria can be defined only after the clinically relevant exposure differences have been identified. This risk-based approach allows bioequivalence assessment to address clinically meaningful differences rather than pharmacokinetic similarity alone.

In clinical practice, therapeutic drug monitoring (TDM) and empiric dose adjustment remain important approaches to controlling exposure to NTI drugs [11,12,13]. However, a single concentration measurement or fixed dosing rule is often insufficient to account for the combined effects of genetic background, organ function, concomitant medications, pathophysiological status, and formulation characteristics on drug exposure [2,14]. Model-informed precision dosing (MIPD) integrates population pharmacokinetic (PopPK) or pharmacokinetic–pharmacodynamic (PK–PD) models with patient characteristics and individual concentration or biomarker data. Bayesian methods can then be used to update individual parameter estimates over time and translate TDM results into quantitative dosing recommendations [15,16,17]. Physiologically based pharmacokinetic (PBPK) models further incorporate drug physicochemical properties, human physiology, metabolic and transport pathways, food and drug interactions, and formulation dissolution characteristics, thereby supporting extrapolation to special populations, virtual bioequivalence trials, and the development of clinically relevant quality standards [18,19,20,21]. Machine learning can identify complex nonlinear relationships in high-dimensional clinical and real-world data and has been applied to blood concentration prediction, dose estimation, treatment-response assessment, and toxicity risk prediction. Reinforcement learning extends this framework by formulating dose optimization as a sequential decision-making problem that is continuously updated as the patient’s clinical state evolves [22].

Although these modeling approaches offer new avenues for the individualized management of NTI drugs, their clinical translation remains limited by the lack of standardized criteria for model quality assessment, insufficient representativeness of training data, limited external generalizability, poor algorithmic interpretability, and a paucity of prospective evidence on clinical outcomes [23,24]. Existing studies [4] have largely addressed the identification criteria for NTI drugs, BE assessment of generic products, international regulatory differences, or the clinical application of individual modeling approaches in isolation. Consequently, the continuum linking drug regulation, formulation performance, individual exposure, and dynamic dose management has not yet been considered within a unified framework [25]. It is therefore necessary to connect premarketing classification, BE assessment, and formulation quality control with postmarketing TDM, model-informed dosing, and risk management [26].

In response to these limitations, this structured narrative review summarizes definitions of narrow therapeutic index (NTI) drugs, their identifying characteristics, and jurisdiction-specific drug lists. It compares the bioequivalence endpoints, study designs, and acceptance criteria used by major regulatory agencies for generic NTI products. It also examines how these regulatory requirements relate to drug-specific clinical risks. The review then evaluates applications of population pharmacokinetic (PopPK), pharmacokinetic–pharmacodynamic (PK–PD), physiologically based pharmacokinetic (PBPK), machine-learning, and hybrid models. These applications include exposure prediction, dose optimization, extrapolation to special populations, virtual bioequivalence assessment, and clinical risk prediction. By distinguishing product-level regulatory assessment, exposure-level quantitative analysis, and patient-level dose management, this review aims to clarify how these components interact throughout the pharmaceutical lifecycle. It also identifies unresolved challenges in regulatory harmonization, model validation, and clinical translation.

## 2. Literature Search Strategy

For this structured narrative review, we searched PubMed, Embase, Web of Science Core Collection, Scopus, and the China National Knowledge Infrastructure (CNKI) on 4 September 2026. We also searched the official websites of relevant regulatory agencies. Literature was eligible if published on or before 30 June 2026. The database searches followed two complementary strategies designed to capture both general and drug-specific evidence. The first combined terms related to narrow therapeutic index (NTI) drugs with terms for regulation, bioequivalence, modelling, or precision dosing. The second paired representative drug names with terms for modelling or precision dosing. Each search strategy was adapted to the controlled vocabulary, search fields, and syntax of the corresponding database. Complete database-specific search strategies are provided in Supplementary Methods S1.

The inclusion criteria were as follows: (1) official documents and studies addressing the definitions, identification criteria, drug lists, or regulatory policies for NTI drugs; (2) studies evaluating bioequivalence, interchangeability, within-formulation variability, or switching-related risks of generic NTI products; (3) studies applying PopPK, PK–PD, PBPK, physiologically based biopharmaceutics modeling (PBBM), machine learning, reinforcement learning, or hybrid models to exposure prediction, dose optimization, extrapolation to special populations, virtual bioequivalence assessment, or clinical risk prediction; and (4) methodological, clinical, and external validation studies directly relevant to these topics. Modeling studies were required to report the data source, modeling approach, validation procedures, and outcomes of clinical or regulatory relevance. Duplicate publications, conference abstracts, commentary articles lacking essential methodological information, studies unrelated to human drug dosing or regulatory evaluation, and nonofficial secondary online sources for which original regulatory documents were available were excluded.

The searches of the five databases yielded 23,927 records, which two reviewers independently screened against the inclusion and exclusion criteria. Disagreements were first resolved through discussion; unresolved cases were adjudicated by a third reviewer. Screening yielded 92 eligible journal articles, and searches of official regulatory agency websites identified 21 additional regulatory documents. In total, the review included 113 sources; the complete selection process is shown in Figure S1.

## 3. Overview of Narrow Therapeutic Index Drugs

The definition and terminology of NTI drugs remain inconsistent across jurisdictions. Different countries and regions use different designations for this drug category. For example, the United States and China commonly use the term “narrow therapeutic index drugs” (NTI drugs), whereas the European Union often uses “NTIDs” (narrow therapeutic index drugs), Japan uses “NTRDs” (narrow therapeutic range drugs), Canada adopts the term “critical dose drugs” (CDDs), and Korea refers to “active substances with a narrow therapeutic index” [4]. Under Title 21 of the Code of Federal Regulations, Section 320.33 (21 CFR 320.33) [27], a drug is considered to have a narrow therapeutic ratio when the median lethal dose (LD_50_) is less than twice the median effective dose (ED_50_), or when the minimum toxic concentration (MTC) in blood is less than twice the minimum effective concentration (MEC). This definition has been widely cited and has influenced the development of related criteria in other jurisdictions. Previous studies [3,8,28] have further suggested that the identification and assessment of NTI drugs should generally consider the following characteristics: (1) close proximity between effective and toxic doses, or between effective and toxic concentrations; (2) a risk of treatment failure when blood concentrations fall below the effective range and of serious adverse effects when concentrations exceed the toxic threshold; (3) a need for TDM based on pharmacokinetic or pharmacodynamic measures; (4) low-to-moderate within-subject variability (≤30%); and (5) relatively small dose adjustments in clinical practice, typically <20%. The indicators described above may support preliminary screening for narrow therapeutic index (NTI) drugs or provide supplementary evidence, but they are generally insufficient to establish NTI status on their own. Classification should also consider the exposure–response relationship, therapeutic window, potential clinical consequences, formulation characteristics, and applicable jurisdiction-specific regulatory requirements. NTI drugs should also be distinguished from highly variable drugs and highly variable drug products. NTI status is a pharmacodynamic and clinical-risk property, reflecting the narrow margin between therapeutic and toxic exposure and the consequences of small exposure changes. By contrast, high variability is a pharmacokinetic and statistical property, typically defined by a within-subject coefficient of variation exceeding 30% for reference-product AUC or C_max_. An NTI drug is not necessarily highly variable, because small exposure changes may remain clinically important despite low AUC or C_max_ variability when its therapeutic window is narrow. Conversely, a highly variable drug does not necessarily have a narrow therapeutic index. Its pharmacokinetic parameters may fluctuate substantially across dosing periods without necessarily causing serious consequences for efficacy or safety. These two dimensions should therefore be distinguished explicitly in clinical practice.

Because regulatory approaches and clinical criteria differ across jurisdictions, international NTI drug lists remain heterogeneous. EMA guidance also acknowledges that NTI drugs cannot be defined using a single universal criterion and should instead be assessed on a case-by-case basis, taking into account the clinical characteristics of the individual drug [29]. A systematic comparison of the classification features and drug lists used for NTI drugs is therefore important for informing regulatory decision-making, guiding generic substitution, and safeguarding clinical use. To preserve distinctions among source types, we classified documents related to narrow therapeutic index (NTI) drugs into three categories. These comprised definitions and designation criteria in regulations or general guidance documents, drug- or product-specific regulatory guidance, and clinical classifications or management recommendations from expert consensus statements. The first category describes the general regulatory framework within the relevant jurisdiction. Drug- or product-specific guidance applies only to the drug, dosage form, or product it addresses. Expert consensus statements, by contrast, reflect professional judgement on clinical risk management. National and regional NTI drug lists are summarized in Table 1. Existing lists show that NTI drugs span multiple therapeutic areas, including hematology, cardiovascular medicine, endocrinology, anti-infective therapy, oncology and immunomodulation, neurology, and respiratory medicine. Warfarin [30,31], digoxin, levothyroxine, tacrolimus, cyclosporine, phenytoin, lithium, and theophylline appear repeatedly across multiple jurisdictions. In contrast, some anti-infective, antidiabetic, and anticancer agents are included only in selected regulatory lists or expert consensus documents, indicating that NTI classification continues to be influenced by regional regulatory practice, perceptions of clinical risk, and differences in the available evidence base.

## 4. Regulatory Requirements and Policy Frameworks for Narrow Therapeutic Index Drugs

Regulatory authorities generally define a generic drug as a medicinal product with the same qualitative and quantitative composition of active substance and the same pharmaceutical form as the reference product, for which bioequivalence has been demonstrated through appropriate bioavailability studies [44]. When applicable regulatory requirements are met, demonstration of bioequivalence generally supports the expectation that the generic and reference products will have comparable efficacy and safety [45]. The core framework used by regulatory authorities to assess bioequivalence for conventional generic drugs is broadly similar across jurisdictions. The area under the concentration–time curve to the last measurable time point (AUC_t_) and the maximum plasma concentration (C_max_) are typically used as the primary pharmacokinetic endpoints. The 90% confidence intervals for the geometric mean ratios of the test and reference products, calculated after logarithmic transformation, are then compared with prespecified bioequivalence acceptance limits. For most drugs, the conventional acceptance range for both AUC_t_ and C_max_ is 80.00–125.00% [4]. For narrow therapeutic index (NTI) drugs, by contrast, even small changes in exposure may affect efficacy or safety. Regulatory frameworks address this risk through different control strategies. Narrower AUC acceptance limits primarily constrain differences in overall exposure between the test and reference products. Whether C_max_ acceptance limits are similarly narrowed depends on the clinical relevance of peak exposure to efficacy, toxicity, or therapeutic drug monitoring. Requirements for the same drug or product may also differ across jurisdictions because of the applicable regulatory documents, dosage form and strength, or reference product. Further differences may arise from fasting or fed conditions, pharmacokinetic endpoints, and statistical criteria. Table 2 separates general provisions from product-specific requirements to clarify the product-level risk-control considerations reflected in these regulatory differences. The EMA specifies that the acceptance range for AUC_t_ should be narrowed to 90.00–111.11% for NTI drugs [46]. The conventional range of 80.00–125.00% may generally be retained for C_max_; however, when C_max_ is particularly relevant to safety, efficacy, or TDM, the acceptance range should also be narrowed to 90.00–111.11%. Similar approaches have been adopted by the WHO [47], Singapore [48], the Gulf Cooperation Council (GCC) [41], and the Egyptian Drug Authority (EDA) [39].

Plasma protein binding (PPB) affects the relationship between total and unbound drug concentrations, but does not directly justify narrower bioequivalence acceptance limits for narrow therapeutic index (NTI) drugs. The 90.00–111.11% acceptance range is intended to constrain differences in absorption rate and extent between the test and reference products. The specific limits are determined primarily by the potential efficacy and safety consequences of altered exposure. In crossover bioequivalence studies, both formulations are compared within the same participants under standardized conditions. If protein binding remains approximately linear across the study concentration range and the unbound fraction is stable, unbound and total exposure ratios are generally similar. Current regulatory assessments therefore usually calculate AUC and C_max_ from total parent-drug concentrations measured in an appropriate biological matrix. When binding is saturable or concentration-dependent, or the unbound fraction is affected by albumin, organ function, metabolites, or concomitant medications, unbound concentrations may provide supplementary evidence. Such measurements may also aid clinical interpretation, but their inclusion in bioequivalence assessment should depend on current drug- and product-specific evidence [49]. This issue warrants particular attention when drug–drug interactions (DDIs) are present. DDIs may alter systemic exposure by affecting protein binding or drug disposition. Bioequivalence assessment should therefore determine whether interaction magnitude depends on the formulation or mode of administration and whether evaluation under specific conditions is warranted. A study in seven healthy participants found that valproate coadministration increased the unbound fraction of phenytoin from 9.6% to 15.6%. The effect involved both displacement from protein-binding sites and metabolic inhibition, suggesting that total concentrations may not fully capture changes in unbound exposure [50]. A four-period crossover study in 18 healthy participants found that voriconazole increased the AUC of immediate-release and extended-release tacrolimus 6.02-fold and 2.62-fold, respectively [51]. This finding suggests that formulation release characteristics may modify the magnitude of a drug interaction. Together, these studies can inform the need for supplementary assessment under specific coadministration conditions and guide therapeutic drug monitoring and dose adjustment. However, current evidence primarily supports drug- or product-specific risk management and does not justify uniform PPB- or DDI-specific bioequivalence acceptance limits for NTI drugs.

The National Institute of Health Sciences (NIHS) of Japan [52] generally retains the conventional bioequivalence acceptance range of 80.00–125.00% for NTI drugs, while applying additional, more stringent criteria. When the initial bioequivalence study includes at least 20 participants (*n* = 10 per group), or when the combined sample size of the initial and supplementary studies is at least 30, and the test and reference products exhibit highly similar dissolution profiles, AUC_t_ and C_max_ may additionally be required to fall within the narrower range of 90.00–111.11%. The Korean Ministry of Food and Drug Safety (MFDS) [53] narrows the acceptance range for AUC_t_ to 90.00–111.11% for NTI drugs, while retaining 80.00–125.00% for C_max_. Health Canada [37] specifies an acceptance range of 90.00–112.00% for AUC_t_, while maintaining 80.00–125.00% for C_max_.

**Figure 1:**
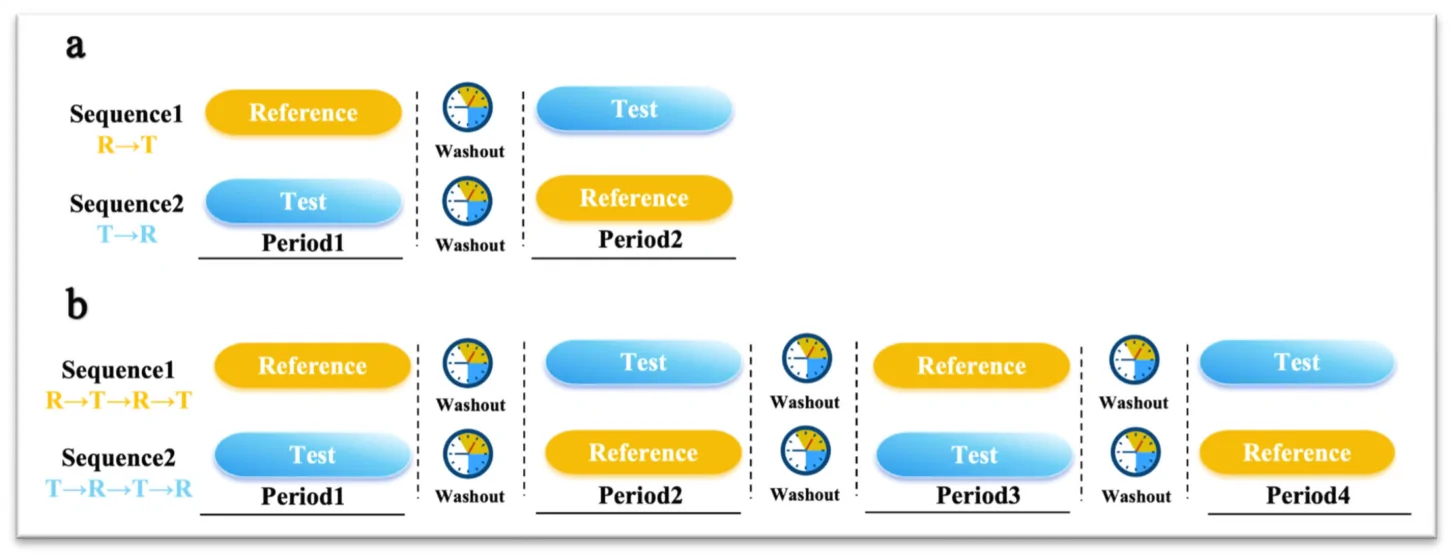
Schematic comparison of crossover designs used in bioequivalence studies of narrow therapeutic index drugs. (**a**) Conventional two-sequence, two-period crossover design, in which each participant receives the reference and test formulations once. (**b**) Fully replicated two-sequence, four-period crossover design, in which each participant receives each formulation twice, allowing the within-subject variability of the reference and test formulations to be estimated separately. R, reference formulation; T, test formulation.

Compared with regulatory approaches based primarily on fixed acceptance ranges, the NMPA/CDE and the FDA apply more complex study designs and multidimensional assessment criteria to selected NTI drugs. Most jurisdictions generally use a standard 2 × 2 two-sequence, two-period crossover design, whereas China [54] and the United States [32] commonly require a fully replicated four-period crossover design for specific NTI drugs (Figure 1). In a 2 × 2 crossover study, participants are randomly assigned to two treatment sequences and receive the test (T) and reference (R) products in successive periods separated by an adequate washout interval. Because each participant receives each product once, participants serve as their own controls, thereby reducing the influence of between-subject variability. This design is simple, relatively short, and less costly, and is therefore widely used in conventional generic-drug BE studies. However, because T and R products are each administered only once, the 2 × 2 design generally permits estimation only of pooled residual variability and does not allow robust, separate estimation of the within-subject standard deviations for T (SWT) and R (SWR). Consequently, this design may provide insufficient evidence when variability is high or when regulatory assessment requires direct comparison of within-subject variability between the two products.

In a fully replicated four-period crossover design, participants are likewise randomized to two treatment sequences, but each participant receives both T and R twice. Repeated administration of each product enables separate estimation of SWT and SWR within the same study and allows direct comparison of their within-subject variability, thereby providing more precise and robust BE evidence for NTI drugs. Its main limitations are the greater number of dosing and washout periods, longer study duration, higher demands on participant adherence, and substantially increased costs and resource requirements. Because even small differences in exposure may have clinical consequences for NTI drugs, China and the United States use replicated designs for selected products to improve the precision and reliability of BE assessment. The preceding overview outlines the basic structures and intended uses of conventional two-period crossover and fully replicated four-period crossover designs. Specific trial conditions, including the study population, administered dose and product strength, fasting or fed conditions, sampling schedule, analyte selection, and safety monitoring, vary by drug and product. They should be determined from the drug’s pharmacokinetic properties, clinical risks, formulation type, and relevant product-specific guidance. Because these conditions differ across drugs and products, and some case-specific assessment details are not fully disclosed publicly, this review does not prescribe uniform trial conditions. Individual protocols should instead be developed for the specific product and in accordance with the regulatory requirements of the relevant jurisdiction.

For these NTI drugs, China and the United States generally assess three criteria simultaneously. First, reference-scaled average bioequivalence (RSABE) is applied. RSABE is an approach that scales bioequivalence assessment to the within-subject variability of the reference product. It is used primarily when the reference product shows substantial within-subject variability. The method evaluates differences between the log-transformed mean exposure metrics of the test and reference products relative to within-subject variability estimated from replicate reference administrations. This comparison determines whether the difference in mean exposure is acceptable relative to the reference product’s own variability. RSABE therefore calibrates average bioequivalence assessment to reference-product variability rather than simply increasing sensitivity to exposure differences. Second, average bioequivalence (ABE) is used as an additional constraint, requiring the 90% confidence intervals for the geometric mean ratios of AUC_t_ and C_max_ to fall within 80.00–125.00%. Third, within-subject variability is compared between T and R, typically requiring the upper bound for the ratio of within-subject standard deviations (σWT/σWR) to be ≤2.5. T and R are considered bioequivalent only when both AUC_t_ and C_max_ satisfy all three criteria: RSABE, ABE, and the within-subject variability comparison. This multidimensional framework evaluates not only the similarity of mean exposure but also whether the test product introduces substantially greater within-subject variability than the reference product, thereby providing additional safeguards for the efficacy and safety of NTI drug therapy. It should be noted that BE requirements for NTI drugs in Taiwan and the Hong Kong Special Administrative Region differ from those in mainland China. The Taiwan Food and Drug Administration (TFDA) [55] retains the conventional acceptance range of 80.00–125.00%, whereas the Drug Office of the Hong Kong Department of Health [42] narrows the acceptance range to 90.00–111.11%.

Differences in regulatory policies for the bioequivalence assessment of NTI drugs largely reflect how individual authorities balance the risks of generic substitution against evidentiary reliability, study feasibility, and drug accessibility. On the one hand, because NTI drugs have narrow therapeutic windows, overly simple study designs or permissive acceptance criteria may fail to detect clinically meaningful differences in exposure. On the other hand, highly complex replicated designs and multiple decision criteria can substantially increase study duration, cost, and operational burden, thereby reducing the efficiency and accessibility of generic NTI drug development. Nevertheless, inconsistent regulatory standards may limit the comparability and cross-jurisdictional applicability of bioequivalence findings [14]. The same generic NTI product may yield different regulatory conclusions across regions because of differences in acceptance ranges, study designs, or statistical methods, complicating global development, multinational submissions, and clinical substitution decisions. Accordingly, a major challenge for international regulatory harmonization is to establish a tiered bioequivalence framework that accounts for therapeutic window, exposure–response relationships, within-subject variability, pharmacokinetic characteristics, and clinical risk, while maintaining both scientific rigor and practical feasibility. However, publicly available information does not fully capture product-specific adjustments made during regulatory review or regulatory considerations that are not publicly documented. Accordingly, assessment of a specific product must integrate the drug’s properties, product characteristics, applicable regulatory policies, and relevant guidance documents.

In addition, some healthcare professionals remain cautious about the safety of generic substitution for NTI drugs [44,56]. Conventional ABE primarily compares mean exposure between the test and reference products and does not fully account for differences in product variability, between-subject variability, or within-subject variability. It therefore provides limited information on whether exposure will remain stable in an individual patient after switching formulations. Population bioequivalence (PBE) and individual bioequivalence (IBE) were proposed in the late 1990s as extensions of average bioequivalence (ABE) [57]. PBE evaluates between-formulation differences in both mean exposure and total variability, theoretically addressing population-level “prescribability.” IBE additionally incorporates within-subject variability and subject-by-formulation interaction, theoretically addressing within-person “switchability.” Both methods characterize pharmacokinetic distributions from a statistical perspective. However, meeting their respective criteria does not, by itself, establish the clinical interchangeability of narrow therapeutic index (NTI) drugs across patients. The limited regulatory use of PBE and IBE partly reflects uncertainties in their statistical interpretation and evidentiary basis. Both rely on composite criteria, allowing differences in formulation means and variance components to offset each other within a single statistic. Results may also be sensitive to the scaling constant, model and distributional assumptions, outliers, and the precision of variance and interaction estimates. Moreover, their decision thresholds have not been adequately validated against clinical outcomes. FDA guidance issued in May 2026 states that IBE is not currently used in regulatory assessment. PBE is used mainly in selected in vitro bioequivalence studies requiring comparisons of both means and variability. For example, FDA’s product-specific guidance for fluticasone propionate nasal spray applies a modified PBE approach to compare the means and total variability of cascade-impactor measurements [58]. By contrast, the replicate-design EQUIGEN study [59] compared branded lamotrigine with two generic products in 49 evaluable patients with epilepsy. Comparisons among the three formulations met the ABE, scaled ABE, and IBE criteria, with no detected subject-by-formulation interaction. The former illustrates the use of PBE for in vitro product-performance comparisons. The latter shows how IBE can assess within-subject variability and interaction effects in replicate pharmacokinetic studies. For in vivo applications, these approaches generally require replicate crossover designs, complex statistical models, larger samples, and stringent data-quality controls. These requirements increase both study costs and operational complexity, partly explaining their limited adoption in routine regulatory standards. Future work should refine the statistical methods and study designs, define appropriate use cases and clinical interpretations, and develop a tiered framework integrating mean exposure, within-subject variability, subject-by-formulation interaction, and clinical risk. Such advances could reduce uncertainty surrounding generic substitution of NTI drugs and support greater international harmonization of bioequivalence regulation. Nevertheless, neither PBE nor IBE can currently replace clinical outcome evidence obtained after actual formulation switching.

**Table 2:** Bioequivalence criteria for NTI generics products across regulatory assessment.

Regulatory Authority	BE Study Design	PK Parameters	Assessment Criteria		
			Conventional Generics	NTI Generics	
			BE Range	AUC_t_ Range	C_max_ Range
EMA [46]	2 × 2 cross-over	AUC_t_, C_max_	80.00–125.00%	90.00–111.11%	Usually 80.00–125.00%; 90.00–111.11% when required
WHO [47]	2 × 2 cross-over	AUC_t_, C_max_	80.00–125.00%	90.00–111.11%	90.00–111.11%
Singapore HSA/ASEAN [48]	2 × 2 cross-over	AUC_t_, C_max_	80.00–125.00%	90.00–111.11%	Usually 80.00–125.00%; 90.00–111.11% when required
Health Canada [37]	2 × 2 cross-over	AUC_t_, C_max_	80.00–125.00%	90.00–112.00%	80.00–125.00%
Japan NIHS/PMDA [52]	2 × 2 cross-over	AUC_t_, C_max_	80.00–125.00%	Usually 80.00–125.00%; 90.00–111.11% when required	Usually 80.00–125.00%; 90.00–111.11% when required
Korea MFDS [53]	2 × 2 cross-over	AUC_t_, C_max_	80.00–125.00%	90.00–111.11%	80.00–125.00%
Saudi Arabia SFDA [41]	2 × 2 cross-over	AUC_t_, C_max_	80.00–125.00%	90.00–111.11%	Usually 80.00–125.00%; 90.00–111.11% when required
Egypt EDA [39]	2 × 2 cross-over	AUC_t_, C_max_	80.00–125.00%	90.00–111.11%	90.00–111.11%
Taiwan TFDA [55]	2 × 2 cross-over	AUC_t_, C_max_	80.00–125.00%	80.00–125.00%	80.00–125.00%
Hong Kong DO [42]	2 × 2 cross-over	AUC_t_, C_max_	80.00–125.00%	90.00–111.11%	90.00–111.11%
China NMPA/CDE [54]	General fully replicated 2-formulation, 2-sequence, 4-period crossover	AUC_t_, C_max_	80.00–125.00%	All three: RSABE; ABE 90% CI, 80.00–125.00%; T/R within-subject variability comparison	All three: RSABE; ABE 90% CI, 80.00–125.00%; T/R within-subject variability comparison
U.S. FDA [32]	Fully replicated 2-formulation, 2-sequence, 4-period crossover	AUC_t_, C_max_	80.00–125.00%	All three: RSABE; ABE 90% CI, 80.00–125.00%; upper confidence bound for σWT/σWR ≤ 2.5	All three: RSABE; ABE 90% CI, 80.00–125.00%; T/R within-subject variability comparison

## 5. Applications of Modeling Approaches to Narrow Therapeutic Index Drugs

**Figure 2:**
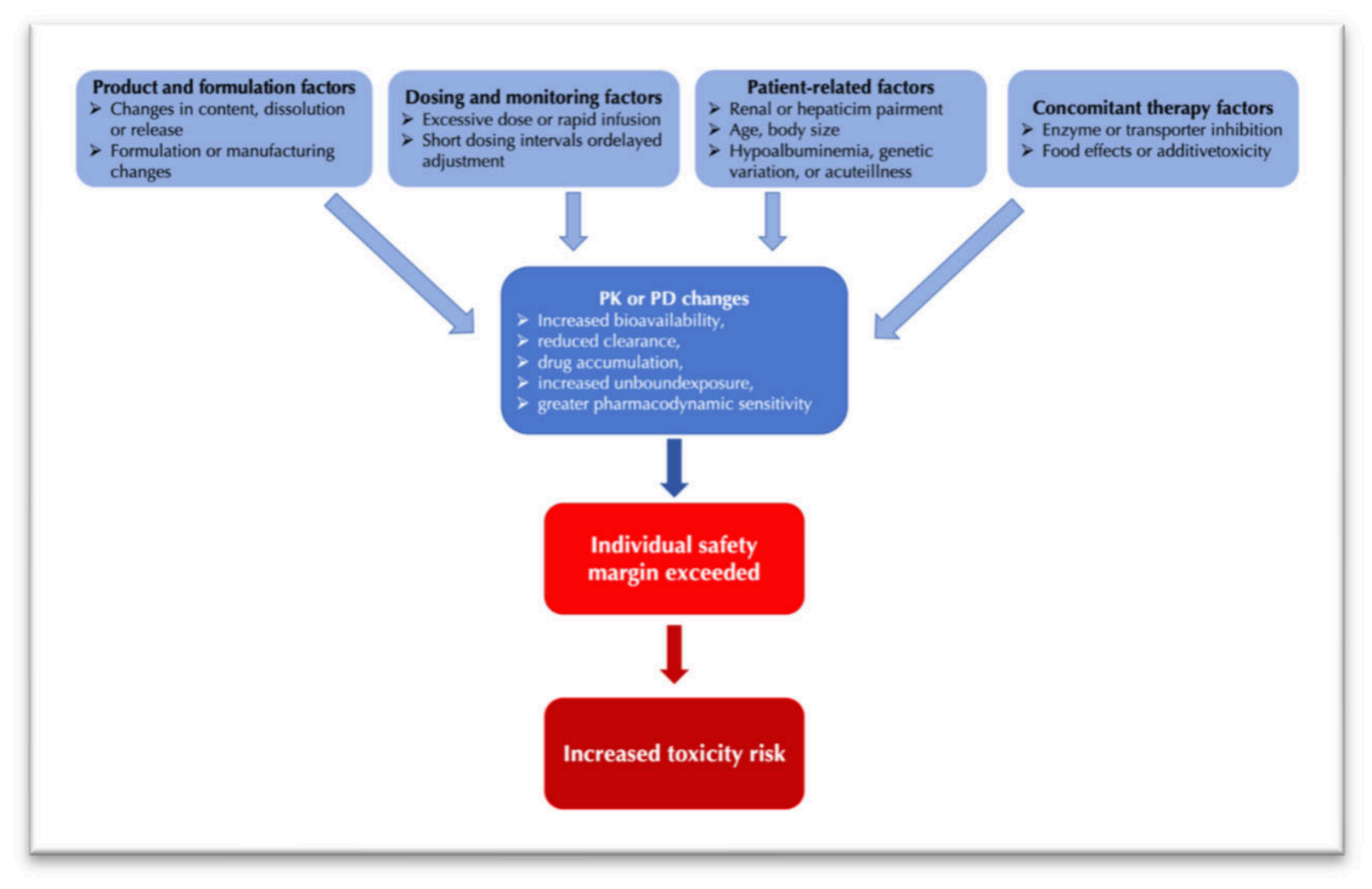
Major pathways leading to increased toxicity risk with narrow therapeutic index drugs. Light blue arrows indicate the effects of different risk factors on PK/PD changes, the dark blue arrow indicates progression to exceeding the individual safety margin, and the red arrow indicates the subsequent increase in toxicity risk.

This review proposes a three-level conceptual framework for the lifecycle risk management of narrow therapeutic index (NTI) drugs. The framework links product-, population-, and individual-level evidence to support decisions across different stages of drug development and clinical use. Its scientific validity and clinical utility, however, require confirmation through further clinical studies and real-world data. At the product level, assessment focuses on NTI classification, formulation performance, and bioequivalence between test and reference products. It examines whether product differences or formulation changes could shift systemic exposure beyond a prespecified acceptable range. At the population level, clinical pharmacokinetic analyses, population pharmacokinetic (PopPK) modelling, and physiologically based pharmacokinetic or biopharmaceutics modelling (PBPK/PBBM) characterize exposure distributions. These approaches examine the effects of formulation factors, drug interactions, and population characteristics. They also identify high-risk scenarios not adequately captured by conventional bioequivalence studies. At the individual level, therapeutic drug monitoring, patient covariates, and Bayesian forecasting guide dose adjustment using measured drug concentrations or pharmacodynamic responses. These three evidence streams serve distinct decision functions, with systemic exposure providing the common quantitative link between them. Premarketing assessments and studies following formulation changes primarily control product-related differences in exposure. Postmarketing surveillance and model updating instead identify and manage between-patient and within-patient exposure variability. Evidence generated during clinical use can, in turn, refine drug interaction management, formulation risk assessment, and product-specific regulatory requirements. Figure 2 summarizes the major factors and pathways that may increase toxicity risk during treatment with NTI drugs.

Precision dosing converts patient-specific information and longitudinal monitoring data into actionable dosing decisions. Current strategies can be grouped according to their decision basis and mode of implementation. These include stratified dosing based on prescribing information and patient characteristics, empirical titration guided by efficacy or toxicity, and therapeutic drug monitoring-guided dose adjustment. Pharmacogenomics-guided dosing and model-informed precision dosing (MIPD) represent two further strategies. For narrow therapeutic index drugs, MIPD integrates population-level priors, patient covariates, and longitudinal concentration or biomarker data. It can estimate individual pharmacokinetic parameters and exposure, compare candidate regimens, and iteratively update dose recommendations through Bayesian forecasting. The appropriate model depends on the decision problem, with population pharmacokinetic models primarily supporting individual parameter estimation and adaptive dose adjustment. Physiologically based pharmacokinetic and biopharmaceutics models (PBPK/PBBM) characterize how physiology, pharmacokinetic drug interactions, and formulation factors shape exposure. Machine-learning models can identify complex nonlinear relationships in high-dimensional data, whereas hybrid models combine approaches to address specific questions (Table 3). MIPD should therefore complement rather than replace integrated clinical judgement. It provides quantitative, iterative support for dose optimization when exposure or response targets are defined, models are adequately validated, and reliable individual-level data are available.

For narrow therapeutic index drugs, model performance should be evaluated against drug-specific target exposure ranges or clinical decision thresholds. Acceptable error margins, including the tolerated magnitude and direction of prediction error, should be prespecified. Evaluation should separately quantify prediction bias and precision, together with the probability that model-informed dosing produces exposure below or above the target range. Statistical measures such as mean absolute error (MAE) and root mean square error (RMSE) can quantify overall prediction error. However, these measures alone cannot establish whether a model is clinically acceptable. This judgement should also consider external validation, sensitivity analyses under clinically relevant scenarios, and prospective clinical evaluation. The relevant decision thresholds should be derived from drug-specific evidence linking exposure to efficacy and toxicity, the therapeutic exposure range, and dose-adjustment objectives.

### 5.1. Applications of Conventional Pharmacokinetic and Population Pharmacokinetic Models to Narrow Therapeutic Index Drugs

Conventional pharmacokinetic (PK) and PopPK models are among the earliest and most extensively studied approaches applied to NTI drugs. Based on concentration–time profiles, these models quantitatively characterize between- and within-subject variability, identify key determinants of drug exposure, and integrate patient characteristics and TDM data into dose-adjustment strategies. Accordingly, conventional PK and PopPK models play an important role in individualized dosing, maintenance of therapeutic efficacy, and long-term safety management for NTI drugs.

Warfarin is a representative NTI drug for which conventional PK and PopPK modeling has been extensively developed. Hamberg et al. [60] established a population PK–PD model based on S- and R-warfarin concentrations, the international normalized ratio (INR), and CYP2C9 and VKORC1 genotypes. The results showed that age and CYP2C9 genotype primarily affected S-warfarin clearance, whereas VKORC1 genotype mainly influenced drug sensitivity. Even when patients exhibited similar early changes in INR, different combinations of covariates could lead to markedly different steady-state anticoagulant responses, indicating that warfarin dose adjustment should not rely solely on empirical interpretation of early INR measurements. Sasaki et al. [61] further combined a population PK–PD model with Bayesian forecasting to develop a maintenance-dose prediction method for Japanese patients. After incorporating individual clearance (CL), the half-maximal effective concentration (EC_50_), and INR, the model-predicted doses showed good agreement with the actual maintenance doses. Wright and Duffull [62] subsequently conducted a prospective validation of the individualized dosing tool TCIWorks. As serial INR measurements were incorporated, the prediction bias for steady-state INR progressively decreased, while the ability to maintain INR within the therapeutic range improved, demonstrating that PopPK modeling combined with Bayesian feedback can directly support clinical dose adjustment. Xue et al. [63] further conducted a randomized controlled trial in Chinese patients using NextDose. Compared with conventional dosing, model-informed dosing increased the time in therapeutic INR range during follow-up and reduced minor bleeding events. Collectively, these studies show that PK and PopPK models for warfarin have evolved from tools for characterizing pharmacokinetic behavior and sources of variability into clinically applicable decision-support systems for dose optimization.

Conventional PK and PopPK studies of antiepileptic drugs have placed greater emphasis on TDM and dose adjustment in special populations, with valproate, phenytoin, and carbamazepine being the most extensively studied examples. Early PopPK studies of valproate showed that body weight, age, and dose were important determinants of its clearance and volume of distribution [64]. Subsequent studies further incorporated its nonlinear protein-binding characteristics to improve the characterization of changes in total and unbound valproate concentrations [65]. Phenytoin exhibits pronounced nonlinear pharmacokinetics, such that small changes in dose may produce substantial changes in blood concentration. Accordingly, research on phenytoin shifted relatively early toward dose individualization based on TDM and Bayesian feedback. In a single-centre retrospective study in Switzerland, Tobler et al. [66] evaluated intravenous phenytoin dosing using prespecified TDM time points and Bayesian forecasting. The proportion of serum concentrations within the prespecified therapeutic range was 63.6%, compared with 34.0% in the conventional-dosing cohort. These findings suggest that TDM combined with Bayesian forecasting may improve target-concentration attainment, but they do not establish better clinical outcomes. Given the retrospective, single-centre design, effectiveness in other populations and clinical settings requires prospective evaluation.

PopPK models of carbamazepine have primarily been used to identify determinants of exposure in special populations. Delgado et al. [67] developed a nonlinear mixed-effects model in pediatric patients with epilepsy and found that body weight, age, dose, and concomitant phenobarbital therapy affected carbamazepine clearance. Punyawudho et al. [68] further demonstrated in older patients that concomitant phenytoin significantly increased carbamazepine clearance; the carbamazepine dose may therefore need to be increased by approximately 23% in older patients receiving phenytoin. By contrast, body weight, age, and routinely measured indices of hepatic and renal function explained little of the pharmacokinetic variability in this population. Overall, the principal value of conventional PK and PopPK models for antiepileptic drugs lies in identifying the sources of exposure variability and translating blood concentration measurements into dose-adjustment strategies tailored to individual patients and specific clinical circumstances.

Conventional PK and PopPK modeling of digoxin began relatively early and has focused primarily on renal function-based dose individualization. Nagaraja et al. [69] reported in Korean patients that digoxin clearance was mainly influenced by creatinine clearance, whereas the volume of distribution was associated with body weight and creatinine clearance. The absorption rate may also have been affected by sex and concomitant medications. Based on data from 142 older patients, Chen et al. [70] found that the interindividual variability in clearance and volume of distribution was 31.6% and 65.8%, respectively. Clearance was influenced by renal function, body weight, type of heart failure, and concomitant use of calcium channel blockers or spironolactone, whereas the volume of distribution was affected by body weight and type of heart failure. Angel et al. [71] subsequently developed a population model using data from 326 patients aged 90 years or older. Their findings similarly identified renal function estimated using the Cockcroft–Gault equation as the principal determinant of digoxin clearance, on the basis of which a corresponding dose-adjustment equation was proposed. These studies indicate that, even in very old patients with multimorbidity, PopPK models can improve the rationality of digoxin dose adjustment by integrating information on renal function, body weight, and concomitant medications.

PopPK modeling is currently the most widely applied approach to model-informed tacrolimus management. Numerous PopPK models have been developed in kidney, liver, heart, and lung transplant recipients [72], incorporating covariates such as CYP3A5 genotype, hematocrit, time after transplantation, age, body weight, transplant type, and concomitant use of CYP3A inhibitors or inducers. Cai et al. [73] developed a PopPK model using 807 whole-blood concentration measurements from 52 Chinese lung transplant recipients. Hematocrit, postoperative time, concomitant voriconazole use, and the CYP3A5*3 genotype were identified as significant determinants of tacrolimus clearance, and dosing optimization strategies were proposed for different clinical scenarios. Such models can not only explain between-patient differences in dose requirements but also be combined with Bayesian feedback to update individual parameter estimates using limited TDM data. A prospective randomized clinical trial conducted by Lloberas et al. [74] showed that, compared with body weight-based dosing and empirical dose adjustment, individualized dosing based on a PopPK model and Bayesian forecasting increased the proportion of kidney transplant recipients who achieved the target trough concentration early after transplantation, shortened the time to target attainment, and reduced the number of dose adjustments. However, no significant improvement in clinical outcomes was observed, suggesting that the current benefits of this approach lie primarily in improving pharmacokinetic target attainment and dose-adjustment efficiency. Its effects on rejection, toxicity, and long-term graft outcomes require further investigation.

In summary, the application of conventional PK and PopPK models to NTI drugs has evolved from the early estimation of pharmacokinetic parameters and identification of sources of variability into clinical decision-support tools that integrate patient characteristics, drug concentrations, and pharmacodynamic measures. These models can clearly characterize the relationships between covariates and drug exposure and continuously update individual parameter estimates through Bayesian feedback, providing strong interpretability and clinical applicability. Although they remain limited in their ability to address high-dimensional nonlinear relationships, complex physiological mechanisms, and extrapolation across populations, conventional PK and PopPK models remain among the most mature modeling frameworks with the strongest foundation for clinical translation in TDM and individualized dosing of NTI drugs.

### 5.2. Applications of Physiologically Based Pharmacokinetic Models to Narrow Therapeutic Index Drugs

Compared with conventional pharmacokinetic models, PBPK models place greater emphasis on describing drug disposition on the basis of physiological structure, drug physicochemical properties, and metabolic and transport mechanisms. Because changes in exposure to NTI drugs are closely associated with clinical outcomes, PBPK modeling offers distinct advantages in predicting complex exposure scenarios, extrapolating to special populations, supporting individualized dosing, evaluating bioequivalence, and establishing clinically relevant quality standards. Current studies indicate that the focus of PBPK applications varies across drugs. These models can be used to quantitatively assess the effects of genetic factors, concomitant medications, and pathophysiological conditions on drug exposure, as well as to link in vitro dissolution characteristics with in vivo exposure and clinical risk. Physiologically based biopharmaceutics modelling (PBBM) provides a quantitative link between in vitro formulation performance and in vivo drug exposure. It integrates formulation composition, critical quality attributes, manufacturing process parameters, dissolution profiles, and clinical pharmacokinetic data. The resulting model can estimate how formulation differences affect drug absorption, C_max_, and AUC. Biopredictive dissolution methods should be developed using formulations with distinct release profiles. The dissolution conditions should discriminate product differences that could alter in vivo exposure. Model predictions should then be validated against the corresponding in vivo pharmacokinetic data. Parameter sensitivity analyses and virtual bioequivalence trials can subsequently define a model-based safe space. This safe space comprises ranges of quality attributes for which predicted exposure remains within product-specific bioequivalence criteria. It can support clinically relevant specifications for dissolution, drug content, and other formulation-performance attributes. When the formulation or manufacturing process changes, pre-change and post-change dissolution profiles can be incorporated into the validated PBBM. Comparisons of predicted AUC and C_max_ can indicate whether formulation performance remains within the established safe space. As a complementary bioequivalence assessment, the dissolution profiles of test and reference products can also be compared. Virtual population simulations can then estimate potential differences in systemic exposure. If routine quality-control methods cannot detect formulation differences with in vivo relevance, the dissolution method may require further refinement. Potential modifications include the dissolution medium, apparatus, agitation conditions, and sampling schedule to improve discriminatory performance. The refined biopredictive method can then support additional quality assessment. Because NTI drugs are particularly sensitive to exposure changes, these applications require stringent evaluation of external predictive performance, parameter uncertainty, and safe-space robustness. Regulatory conclusions should therefore be reached on a product-specific basis.

Studies of warfarin illustrate the dual value of PBPK modeling in clinical dosing and formulation assessment. Cheng et al. [75] found that a conventional PBPK framework could not adequately characterize warfarin pharmacokinetics over 15 days following a single oral dose, particularly the terminal disposition of S-warfarin. To improve predictive performance, the investigators incorporated a target-mediated drug disposition mechanism and accounted for factors such as CYP2C9 genotype and concomitant medications. This approach provided a more complete characterization of S- and R-warfarin exposure and a mechanistic basis for dose selection. Geng et al. [76] subsequently developed an S-warfarin PBPK–PD model that integrated CYP2C9 and VKORC1 genotypes and drug interactions within a unified framework. The model was used to predict the effects of drug–drug and drug–gene interactions on warfarin pharmacokinetics and INR and to support assessment of dose-adjustment requirements during concomitant administration of CYP2C9 inducers. For formulation assessment, Cheng et al. [77] developed a PBPK absorption model for warfarin sodium tablets under fasting and fed conditions to predict acceptable dissolution ranges within which a test product would remain bioequivalent to the reference product under different dosing conditions. Li et al. [78] further combined PBPK modeling with virtual bioequivalence trials to evaluate clinically relevant dissolution ranges and formulation safety margins for warfarin sodium tablets. Collectively, PBPK research on warfarin has expanded from mechanistic characterization of complex in vivo disposition to the prediction of drug interactions, bioequivalence assessment, and the development of clinically relevant quality standards.

Among conventional antiepileptic drugs, PBPK applications have developed distinct emphases according to the pharmacokinetic characteristics of each agent. Phenytoin exhibits pronounced nonlinear pharmacokinetics, and its PBPK models have therefore been used primarily to predict complex exposure profiles and drug interactions. Rodriguez-Vera et al. [79] developed a phenytoin PBPK model using data from 15 studies in healthy participants. The model adequately characterized the pharmacokinetics of single and multiple oral and intravenous doses across the 248–900 mg dose range and incorporated food effects and dose-dependent changes. It also successfully predicted interactions between phenytoin and fluconazole, omeprazole, and itraconazole, indicating that PBPK modeling can support exposure assessment and dose selection in high-risk combination-therapy settings. PBPK studies of carbamazepine have focused more extensively on virtual BE assessment and the establishment of clinically relevant quality standards. Pawar et al. [80] incorporated biorelevant dissolution data from adults and children into a PBPK model and found that in vitro dissolution results showed good in vivo predictive performance when 500 mL and 200 mL of biorelevant medium were used for adults and children, respectively. Virtual BE trials further supported bioequivalence between the reference and generic products in both populations. Building on this approach, Wang et al. [81] integrated PBPK modeling, in vitro dissolution profiles, and clinical pharmacokinetic data to develop and validate a virtual BE assessment method for 100 mg carbamazepine tablets. The study proposed dissolution specifications of Q_60_ min ≥ 80% and 50% ≤ Q_15_ min ≤ 85%, together with an assay range of 95–105%. These findings indicate that PBPK research on carbamazepine has progressed beyond virtual BE assessment toward the definition of clinically relevant quality attributes. PBPK studies of valproate have primarily addressed dose optimization and exposure assessment in special populations. Ogungbenro et al. [82] developed early PBPK models for adults and children that adequately predicted plasma concentrations after intravenous and oral administration and were used to explore appropriate dosing regimens. Karatza et al. [83] subsequently incorporated albumin-binding mechanisms into the model, enabling simultaneous prediction of total and unbound valproate concentrations. The results suggested that children with normal albumin levels were more likely to achieve the conventional TDM range at doses of 30–45 mg/kg/day, whereas measured blood concentrations in children with hypoalbuminemia might not accurately reflect actual drug exposure. A PBPK model developed by van der Heijden et al. [84] yielded similar findings, indicating that valproate generally achieved therapeutic concentrations but that monitoring of unbound valproate should receive greater attention in patients with low albumin levels. Thus, PBPK research on valproate has extended beyond general simulation of pharmacokinetic processes to support the interpretation of exposure and optimization of monitoring strategies under specific physiological conditions.

PBPK models can integrate mechanisms related to CYP3A4/5 metabolism, food effects, formulation release, and drug–drug interactions to predict changes in tacrolimus exposure across genotypes, concomitant therapies, and dosing conditions. Loer et al. [85] developed a whole-body PBPK model using 37 whole-blood concentration–time datasets from 911 healthy participants. The model was subsequently applied to predict food effects and drug interactions involving voriconazole, itraconazole, rifampicin, and other agents, with most predicted exposure ratios falling within twofold of the observed values. For formulation assessment, PBBM can further link in vitro dissolution, gastrointestinal absorption, and systemic exposure. Bi et al. [86] developed a PBBM and biopredictive dissolution method for 5 mg extended-release tacrolimus capsules and used virtual BE trials to establish a bioequivalence safe space. This approach could identify formulations that satisfy conventional quality-control criteria but may still carry a risk of bioinequivalence. PBPK and PBBM therefore provide mechanistic support for evaluating drug interactions, formulation-related differences, and virtual BE.

Overall, the application of PBPK models to NTI drugs has expanded from simple prediction of blood concentrations to the evaluation of genetic effects and drug interactions, dose optimization in special populations, virtual BE trials, and the establishment of clinically relevant quality standards. Although the specific focus varies across drugs, their common value lies in using mechanistic information to link drug properties, physiological characteristics, formulation performance, and clinical exposure, thereby providing a quantitative basis for predicting high-risk scenarios that cannot be adequately evaluated in conventional clinical trials. PBPK modeling has therefore become an important approach to precision dosing, generic drug development, and lifecycle risk management for NTI drugs.

### 5.3. Applications of Machine-Learning Models to Narrow Therapeutic Index Drugs

Machine-learning models have been applied to NTI drugs primarily for predicting drug exposure, optimizing dosing, and assessing risks related to efficacy and safety. Compared with conventional pharmacokinetic models, machine learning can simultaneously accommodate high-dimensional covariates, complex nonlinear relationships, and heterogeneity in real-world data. Accordingly, it has been increasingly explored in recent years for the individualized management of anticoagulants, anti-infective agents, and antiepileptic drugs.

The extensive accumulation of TDM and clinical outcome data for warfarin has provided a strong foundation for the development of machine-learning models. Existing studies have focused primarily on predicting changes in INR, estimating maintenance doses, and developing sequential dose-adjustment strategies. Lee et al. [87] used data from 19,719 hospitalized patients across three hospitals to develop a warfarin-associated prothrombin time–international normalized ratio (PT-INR) prediction system based on a hybrid architecture combining dense neural networks and recurrent neural networks. Using dosing and INR data from the preceding 4 days, the model predicted PT-INR on day 5 and generated an individualized dose–PT-INR response table. In an external hospital dataset, its predictive accuracy was slightly higher than that of medical experts (84.0% vs. 81.9%, *p* = 0.014). Kim et al. [88] used electronic health record data from hospitalized Korean patients to develop a model for predicting the warfarin maintenance dose at discharge. The development and external validation cohorts included 3168 and 891 patients, respectively. In internal validation, the mean absolute error (MAE) was 0.9 for both the XGBoost and artificial neural network models and 1.0 for both random forest and linear regression, compared with 1.3 for physician judgment. These findings suggest that machine learning can improve the accuracy of maintenance-dose prediction based on information collected early during hospitalization. Dryden et al. [89] compared penalized linear regression, k-nearest-neighbor regression, random forest, gradient-boosting regression, multivariate adaptive regression splines, and ensemble models for predicting INR after warfarin treatment in cardiac surgery patients. Random forest performed best among patients with a target INR of 2.0–3.0, whereas the ensemble model performed best among those with a target INR of 2.5–3.5. After implementation of the model in the clinical workflow, the proportion of patients who achieved the target INR at discharge increased from 47.5% to 61.1%, indicating potential clinical utility. Beyond static prediction, machine learning has also been applied to sequential dose decision-making. Nelson et al. [90] used data from warfarin-treated patients enrolled in four large randomized trials of atrial fibrillation to develop a reinforcement-learning model based on a semi-Markov decision process and batch-constrained deep Q-learning. The model dynamically recommended dosing strategies intended to maintain INR within the range of 2.0–3.0. The findings suggested that dose adjustments consistent with the algorithm’s recommendations may increase the proportion of time spent within the therapeutic range. Overall, machine-learning research on warfarin has progressed from single-dose or INR prediction toward sequential dose adjustment and clinical decision support, although its clinical benefits require further confirmation in prospective studies.

Unlike warfarin studies, which focus primarily on controlling anticoagulation intensity, machine-learning research on vancomycin has concentrated on exposure target attainment, initial dose optimization, and identification of nephrotoxicity risk. This research has progressed from early prediction of dose requirements and trough concentrations to the prediction of the area under the concentration–time curve (AUC) and pretreatment risk assessment. Tang et al. [91] developed separate machine-learning models to predict steady-state trough concentrations and AUC_0–24_. External validation showed that these models outperformed previously developed PopPK models. Virtual trials further indicated that machine learning–guided dosing increased the proportion of pediatric patients achieving the target exposure range, suggesting potential for dose optimization in populations with substantial pharmacokinetic variability and limited sampling opportunities. Lee et al. [92] used data from hospitalized patients to develop and externally validate OPTIVAN, an initial-dose optimization model based on gradient boosting machines, random forests, support vector machines, and XGBoost. The model was designed to predict whether an initial dosing regimen would achieve the target AUC_24_/MIC range of 400–600. Hu et al. [93] applied a random forest model to predict steady-state trough concentrations and reported greater accuracy in external validation than the comparator PopPK model. Using 2205 vancomycin treatment records from intensive care unit patients, Ghanbari et al. [94] developed the deep-learning platform Nephrocast-V for trough concentration prediction. Its predictive performance was comparable to estimates generated by pharmacists using Bayesian dose-optimization software. In addition to exposure prediction, machine learning has been applied to vancomycin safety assessment. Mizuno et al. [95] used retrospective clinical data and multiple algorithms, including least absolute shrinkage and selection operator regression, support vector machines, naïve Bayes, decision trees, random forests, and AdaBoost, to develop a model for predicting vancomycin-associated nephrotoxicity, incorporating vancomycin AUC as a predictor. Collectively, machine-learning research on vancomycin has begun to establish an application pathway encompassing initial dose selection, exposure prediction, and toxicity risk assessment. However, the available evidence remains derived mainly from retrospective studies and evaluations of model performance, and whether these approaches improve clinical outcomes requires further confirmation.

Antiepileptic drugs have also been a major focus of machine-learning applications, with research primarily addressing blood concentration prediction and treatment-response assessment. Yao et al. [96] compared multiple machine-learning algorithms using real-world trough concentration monitoring data from Chinese adults with epilepsy and found that the extremely randomized trees model performed best. By integrating information on dose, concomitant medications, and laboratory indices, the model improved the accuracy of steady-state valproate trough concentration prediction. Chen et al. [97] developed a valproate trough concentration prediction model in pediatric patients by integrating gradient-boosting regression trees, random forest regression, and support vector regression, and validated it in two external datasets. The model showed good predictive accuracy and goodness of fit, with platelet count and daily dose identified as key predictors, suggesting that it may support valproate dose adjustment in children. Machine learning has also been used to predict response to antiepileptic treatment. Hakeem et al. [98] developed a deep-learning model using multinational cohorts of patients with newly diagnosed epilepsy to predict outcomes after the first antiepileptic drug treatment. Although the model showed moderate predictive performance in the pooled cohort, its external validation performance was limited, indicating that cross-population generalizability requires further improvement. The number of pretreatment seizures, psychiatric comorbidity, electroencephalographic findings, and neuroimaging results were the principal predictors. Despite the model’s limited overall discrimination, the study suggested that integration of multicenter data may improve the stability and generalizability of treatment-response prediction. Using TDM data from Seoul National University Hospital, Chung et al. [99] compared machine-learning models with previously published PopPK models for predicting concentrations of carbamazepine, phenobarbital, phenytoin, and valproate. AdaBoost, XGBoost, and random forest models showed better overall predictive performance than the published PopPK models for carbamazepine, phenytoin, and valproate. Time since the last dose was among the important predictors. Collectively, these studies indicate that machine learning can effectively accommodate sparse sampling, high-dimensional covariates, and complex nonlinear relationships in antiepileptic drug therapy, thereby extending the scope of conventional TDM models.

Machine-learning models provide a new technical approach to tacrolimus concentration prediction. Tang et al. [100] compared multiple linear regression with eight machine-learning methods in 1045 Chinese kidney transplant recipients and found that the regression tree model outperformed the conventional linear model in dose prediction. Zhang et al. [101] compared several machine-learning and deep-learning algorithms using 5439 dosing records from 584 kidney transplant recipients. The TabNet model achieved good predictive performance in the test set, suggesting that high-dimensional clinical variables can support individualized tacrolimus dose estimation.

Available studies of narrow therapeutic index drugs indicate that PBPK and machine-learning models generate different forms of evidence and address different questions. PBPK studies integrate drug properties, physiological parameters, and formulation information to predict drug interactions, exposure in special populations, and formulation performance. Their outputs typically include mechanistic interpretation, extrapolative prediction, virtual bioequivalence assessment, and support for setting formulation quality specifications. Machine-learning studies instead use therapeutic drug monitoring or other clinical data to predict drug concentrations, dose requirements, therapeutic response, and toxicity risk. Evaluation typically focuses on prediction error, discriminative performance, performance in external validation, or simulated target-attainment rates. Few studies have directly compared these approaches using the same drug, dataset, endpoint, and validation population. This evidence gap precludes robust conclusions about their relative performance. Method selection should therefore follow the research question rather than an assumed hierarchy between approaches. PBPK is generally more suitable for mechanistic extrapolation, drug-interaction prediction, and formulation assessment. Machine learning is better suited to well-defined prediction tasks supported by adequate and representative clinical data. The complementary evidence generated by the two approaches also provides a rationale for developing hybrid models.

In summary, the application of machine-learning models to NTI drugs has expanded from simple dose or concentration prediction to dynamic dosing decisions, treatment-response assessment, and early warning of adverse-effect risks. Their principal advantage lies in the ability to identify complex relationships from large-scale real-world data that are difficult to characterize using conventional models. However, model performance is highly dependent on data quality, sample representativeness, and external validation. Most available studies remain retrospective and focus primarily on predictive accuracy, whereas evidence regarding clinical outcomes, model interpretability, cross-center generalizability, and integration into routine clinical workflows remains limited. Future research should therefore strengthen multicenter external validation and prospective clinical evaluation and clarify the incremental clinical value of machine-learning models over conventional dose-management approaches.

### 5.4. Applications of Hybrid Models to Narrow Therapeutic Index Drugs

Hybrid models integrate mechanistic models with data-driven approaches, combining pharmacological interpretability with the predictive capacity required for complex clinical data. Existing studies have primarily combined PopPK or PK–PD models with machine-learning methods. The former provide mechanistic information on drug clearance, distribution, and exposure–response relationships, whereas the latter identify high-dimensional covariates and complex nonlinear associations. Their applications have gradually expanded from blood concentration prediction to dynamic dose adjustment, treatment-response assessment, and individualized therapy in special populations.

Warfarin was among the first drugs investigated using hybrid modeling approaches [102]. Anzabi et al. [103] first used a PK–PD model to simulate dose–response trajectories in virtual patients and then trained a reinforcement-learning model on the simulated data to dynamically optimize dosing regimens. The model outperformed several dosing strategies commonly used in clinical practice in the virtual patient population. This study demonstrated that mechanistic models can provide reinforcement learning with a pharmacologically plausible training environment, enabling warfarin dose optimization to progress from one-time maintenance-dose prediction toward sequential dose-adjustment decisions.

In the field of anti-infective therapy, hybrid modeling studies have focused primarily on vancomycin and voriconazole. Vancomycin studies commonly use pharmacokinetic parameters estimated by PopPK models as inputs to machine-learning algorithms to improve exposure prediction early in treatment. Li et al. [104] incorporated vancomycin clearance predicted by an existing PopPK model into a machine-learning model. The hybrid model produced lower absolute and relative errors than the PopPK model alone, indicating improved prediction of individual clearance in Chinese adults. Wang et al. [105] further combined PopPK-derived parameters with clinical characteristics in a machine-learning model for older patients. The final model achieved a coefficient of determination (R^2^) of 0.656; 81.82% of predictions differed from the observed concentrations by no more than 5 mg/L, and 76.62% had a relative error of no more than 30%, demonstrating that the hybrid approach could improve vancomycin concentration prediction. Chen et al. [106] compared the predictive performance of PopPK, Bayesian, machine-learning, and hybrid models in patients with sepsis. Before any measured vancomycin concentrations were available, the hybrid model outperformed the PopPK and Bayesian models, reducing the mean absolute percentage error by 58% and 17%, respectively. Once concentration-monitoring data became available, however, the Bayesian model performed best, with a mean absolute percentage error of 13.37%, compared with 68.17%, 34.17%, and 28.52% for the PopPK, machine-learning, and hybrid models, respectively. These findings indicate that the relative advantages of different models depend on data availability. Hybrid models may be more suitable for initial dose prediction early in treatment, before blood concentration measurements are available, whereas Bayesian models may be more effective for subsequent dose adjustment once individual concentration data have been obtained. Studies of voriconazole further demonstrate the value of combining PopPK and machine-learning approaches in TDM. Liu et al. [107] incorporated PopPK-derived parameters into machine-learning models for older patients. An ensemble model comprising XGBoost, random forest, and CatBoost performed best, achieving an R^2^ of 0.828, and could support real-time prediction of voriconazole concentrations and individualized dosing. Shen et al. [108] first developed a PopPK model in immunodeficient children younger than 2 years and then entered individual clearance, volume of distribution, and clinical characteristics into an XGBoost model. External validation yielded an R^2^ of 0.75, suggesting that this approach may support initial dose selection before TDM results become available.

Hybrid modeling studies of antiepileptic drugs have focused primarily on predicting valproate concentrations and evaluating treatment response to combination therapy in pediatric patients. The general approach is to use PopPK models to provide pharmacokinetic parameters and mechanistic constraints, while machine-learning methods integrate complex covariates such as genotype, biochemical indices, and concomitant medications. Zhu et al. [109] used two previously published PopPK models of valproate to generate a large simulated dataset based on a one-compartment model and Monte Carlo simulation. XGBoost and Shapley additive explanations (SHAP) were then applied to integrate covariates from the different PopPK models and develop a model for predicting steady-state drug concentrations. In the validation set, the mean absolute error was 2.4 mg/L, and 98.85% of predicted values were within ±20% of the observed concentrations. Daily dose, dosing time, CYP2C19 genotype, albumin concentration, and body weight were the principal predictors. These findings indicate that the hybrid framework can integrate information from different PopPK models and support individualized valproate dosing. Ma et al. [110] included 43 variables, including PopPK-derived parameters, in a study of older patients with epilepsy. Recursive feature elimination was used for variable selection, and CatBoost, LightGBM, and random forest models were combined using weights of 7:2:1. Incorporation of PopPK parameters improved the predictive performance of each individual machine-learning model, and the final ensemble model achieved an R^2^ of 0.74. A reduced set of 11 selected variables produced predictive performance comparable to that of the full-variable model, suggesting that PopPK parameters may not only improve model accuracy but also reduce redundant variables and enhance clinical implementability. The application of hybrid models has also expanded from concentration prediction to treatment-response assessment. Damnjanović et al. [111] included 71 children receiving combination therapy with valproate, lamotrigine, and levetiracetam. Separate PopPK models were developed for the three drugs, after which the estimated pharmacokinetic parameters and patient characteristics were entered into principal component analysis, factor analysis of mixed data, and random forest models to identify factors associated with seizure control. The pharmacokinetics of all three drugs were adequately described by one-compartment models with first-order absorption and elimination. Random forest showed the best performance for seizure prediction; antiepileptic drug concentrations and body weight were the most important predictors, whereas sex contributed relatively little. This study demonstrates that combining PopPK and machine-learning approaches can support not only the prediction of drug exposure but also the linkage of drug concentrations to clinical response.

Applications of hybrid models to tacrolimus therapy are also gradually emerging. Li et al. [112] combined PopPK and machine-learning approaches to predict tacrolimus trough concentrations during the early postoperative period in Chinese adult liver transplant recipients. Wang et al. [113] developed a PopPK model in 127 Chinese kidney transplant recipients and incorporated its outputs, together with postoperative time, CYP3A5 genotype, hematocrit, and other clinical variables, into machine-learning models. The PopPK-informed XGBoost model showed the best performance in the test set, with lower prediction errors than the standalone PopPK model and the other machine-learning models. These hybrid approaches indicate that mechanistic and data-driven models are complementary rather than mutually substitutive. By integrating model-derived outputs, clinical variables, and algorithmic learning, they may improve concentration prediction under conditions of sparse tacrolimus TDM data.

Overall, the advantages of hybrid models in NTI drug therapy are becoming increasingly evident. Mechanistic models provide pharmacologically plausible parameters, structures, and constraints, whereas machine-learning methods facilitate the analysis of high-dimensional covariates, complex nonlinear relationships, and heterogeneous real-world data. Their integration can improve early-treatment concentration prediction and dose optimization in special populations and may further support dynamic dosing and clinical outcome assessment. However, the appropriate model may differ across stages of treatment. When individual monitoring data are unavailable, hybrid models may support initial exposure prediction; once blood concentration data become available, Bayesian feedback models may be better suited to sequential parameter updating and dose adjustment. Future studies should therefore select integration strategies according to the clinical question, data type, and stage of treatment and should strengthen external validation, prospective evaluation, and validation against clinical outcomes to determine the incremental value of hybrid models over standalone approaches.

## 6. Conclusions

Narrow therapeutic index drugs remain challenging across regulatory evaluation, generic substitution, and clinical dose management because small changes in exposure may compromise efficacy or safety. Current regulatory frameworks differ substantially in drug classification, bioequivalence study design, acceptance criteria, and variability assessment, limiting cross-jurisdictional consistency. PopPK models coupled with Bayesian forecasting currently have a comparatively mature evidence base for therapeutic drug monitoring and individualized dose adjustment. PBPK models primarily support mechanistic extrapolation, drug-interaction prediction, virtual bioequivalence assessment, and evaluation of formulation performance. Machine-learning and hybrid models show potential for predicting exposure and dose requirements, but current evidence comes mainly from retrospective analyses and simulation studies. Their prospective clinical benefit and suitability for regulatory use therefore remain to be established. Future efforts should focus on harmonizing risk-based bioequivalence requirements, standardizing model development and validation, and integrating regulatory, formulation, pharmacokinetic, and patient-level information throughout the drug lifecycle. Such an integrated framework may improve the reliability of generic substitution and support safer, more precise use of narrow therapeutic index drugs.

## Figures and Tables

**Table 1:** National and regional lists of narrow therapeutic index drugs.

Drug	US FDA [32]	EU EMA [29,33]	Japan PMDA [34,35]	Korea MFDS [36]	Health Canada [37,38]	Egypt EDA [39]	Jordan JFDA [40]	Saudi Arabia SFDA [41]	Hong Kong DO [42]	Chinese Expert Consensus [43]
1. Blood and blood-forming organs										
Warfarin	NTID		NTRD	NTID	CDD	NTID	NTRD		NTRD	Strongly recommended
Acenocoumarol		NTID								
2. Cardiovascular system										
Digoxin	NTID		NTRD	NTID	CDD	NTID			NTRD	Strongly recommended
Digitoxin			NTRD	NTID		NTID			NTRD	
Quinidine			NTRD	NTID		NTID	NTRD		NTRD	Strongly recommended
Disopyramide			NTRD	NTID		NTID	NTRD		NTRD	Recommended
Flecainide				NTID	CDD	NTID			NTRD	
Procainamide			NTRD	NTID		NTID	NTRD		NTRD	Recommended
Clonidine			NTRD	NTID		NTID	NTRD		NTRD	Recommended
Guanethidine			NTRD	NTID		NTID	NTRD		NTRD	
Prazosin			NTRD	NTID		NTID	NTRD		NTRD	Recommended
Minoxidil				NTID		NTID	NTRD		NTRD	
Isoprenaline/Isoproterenol			NTRD	NTID		NTID	NTRD		NTRD	Recommended
Isoetharine				NTID		NTID	NTRD		NTRD	
Aprindine			NTRD	NTID		NTID			NTRD	
Metaproterenol/Orciprenaline				NTID		NTID	NTRD		NTRD	
3. Genitourinary system and sex hormones										
Ethinyl estradiol/Progesterone			NTRD	NTID		NTID	NTRD		NTRD	Recommended
4. Hormonal preparations, excluding sex hormones and insulins										
Levothyroxine	NTID	NTID		NTID		NTID		NTID	NTRD	Strongly recommended
Liothyronine	NTID			NTID						
5. Anti-infective										
Clindamycin			NTRD	NTID		NTID	NTRD		NTRD	Recommended
Chloramphenicol									NTRD	
Amphotericin B										Recommended
Vancomycin										Strongly recommended
Voriconazole										Strongly recommended
Teicoplanin										Strongly recommended
6. Antineoplastic and immunomodulating agents										
Tacrolimus	NTID	NTID	NTRD	NTID	CDD	NTID		NTID	NTRD	Strongly recommended
Cyclosporine	NTID	NTID	NTRD	NTID	CDD	NTID			NTRD	Strongly recommended
Sirolimus	NTID	NTID		NTID	CDD	NTID		NTID	NTRD	Strongly recommended
Everolimus	NTID	NTID		NTID				NTID		Recommended
Methotrexate			NTRD	NTID		NTID			NTRD	Strongly recommended
Mycophenolate mofetil										Strongly recommended
Busulfan										Recommended
Paclitaxel										Recommended
Mercaptopurine										Strongly recommended
7. Nervous system										
Phenytoin	NTID		NTRD	NTID	CDD	NTID	NTRD		NTRD	Strongly recommended
Valproate/Valproic acid/Divalproex	NTID		NTRD	NTID		NTID	NTRD	NTID	NTRD	Strongly recommended
Carbamazepine	NTID		NTRD	NTID		NTID	NTRD	NTID	NTRD	Strongly recommended
Lithium	NTID		NTRD	NTID	CDD	NTID	NTRD		NTRD	Strongly recommended
Primidone			NTRD	NTID		NTID	NTRD			Recommended
Phenobarbital			NTRD	NTID		NTID			NTRD	
Ethosuximide			NTRD	NTID		NTID				
Clonazepam			NTRD	NTID		NTID			NTRD	
Zonisamide			NTRD	NTID		NTID			NTRD	
Levodopa/Carbidopa									NTRD	
8. Respiratory system										
Aminophylline			NTRD	NTID		NTID	NTRD		NTRD	Strongly recommended
Theophylline	NTID		NTRD	NTID	CDD	NTID	NTRD		NTRD	Strongly recommended
Choline theophylline			NTRD	NTID					NTRD	Recommended
Diprophylline/Dyphylline			NTRD	NTID		NTID	NTRD		NTRD	Recommended
Oxtriphylline				NTID		NTID	NTRD			
Proxyphylline			NTRD	NTID					NTRD	
9. Alimentary tract and metabolism/antidiabetic agents										
Sulfonylurea compounds			NTRD	NTID		NTID			NTRD	
Glibenclamide									NTRD	
Gliclazide									NTRD	
Tolbutamide									NTRD	
Tolazamide									NTRD	
Acetohexamide									NTRD	
Glybuzole			NTRD						NTRD	
10. Antigout preparations										
Colchicine		NTID						NTID		

**Table 3:** Comparison of modeling approaches applied to NTI drugs.

Model	PK/PopPK	PBPK	Machine Learning	Hybrid Models
Key characteristics	Exposure and variability from concentration–time data	Mechanistic simulation of in vivo disposition	Exposure–outcome learning from high-dimensional clinical data	Integration of mechanistic and data-driven models
Common methods	Compartmental and nonlinear mixed-effects models; Bayesian forecasting	Whole-body PBPK; IVIVE; virtual populations and virtual BE	Random forest; XGBoost; neural networks; reinforcement learning	PopPK/PK–PD/PBPK combined with machine learning
Primary applications	TDM-guided individualized dosing	DDI prediction; special-population extrapolation; formulation and BE assessment	Concentration and dose prediction; efficacy and toxicity assessment	Early exposure prediction; dynamic dosing; outcome assessment
Strengths	Interpretable; mature; widely used clinically	Mechanistic; supports extrapolation across populations and scenarios	Handles nonlinear relationships and heterogeneous data	Balances mechanistic interpretability and predictive performance
Limitations	Limited for high-dimensional nonlinearity and complex extrapolation	Parameter-intensive; complex development and validation	Limited interpretability and external generalizability	Methodologically complex; no standardized validation framework

Abbreviations: PK, pharmacokinetics; PopPK, population pharmacokinetics; PBPK, physiologically based pharmacokinetics; PK–PD, pharmacokinetics–pharmacodynamics; TDM, therapeutic drug monitoring; DDI, drug–drug interaction; BE, bioequivalence; IVIVE, in vitro–in vivo extrapolation.

## Data Availability

The raw data supporting the conclusions of this article will be made available by the authors on request.

## Supplementary Materials

The following supporting information can be downloaded at: https://www.mdpi.com/article/10.3390/pharmaceutics18091190/s1.

## Abbreviations

The following abbreviations are used in this manuscript:

ABE	Average bioequivalence
AdaBoost	Adaptive boosting
ASEAN	Association of Southeast Asian Nations
AUC	Area under the concentration–time curve
AUC_0–24_	Area under the concentration–time curve from 0 to 24 h
AUC_t_	Area under the concentration–time curve to the last measurable time point
BE	Bioequivalence
CatBoost	Categorical boosting
CDD	Critical dose drug
CDE	Center for Drug Evaluation
CFR	Code of Federal Regulations
CI	Confidence interval
CL	Clearance
C_max_	Maximum plasma concentration
CNKI	China National Knowledge Infrastructure
CYP	Cytochrome P450
DDI	Drug–drug interaction
DO	Drug Office of the Hong Kong Department of Health
EC_50_	Half-maximal effective concentration
ED_50_	Median effective dose
EDA	Egyptian Drug Authority
EMA	European Medicines Agency
EU	European Union
FDA	U.S. Food and Drug Administration
GCC	Gulf Cooperation Council
HSA	Health Sciences Authority
IBE	Individual bioequivalence
INR	International normalized ratio
IVIVE	In vitro–in vivo extrapolation
JFDA	Jordan Food and Drug Administration
LD_50_	Median lethal dose
LightGBM	Light gradient-boosting machine
MAE	Mean absolute error
MEC	Minimum effective concentration
MFDS	Ministry of Food and Drug Safety
MIC	Minimum inhibitory concentration
MIPD	Model-informed precision dosing
MTC	Minimum toxic concentration
NIHS	National Institute of Health Sciences
NMPA	National Medical Products Administration
NTI	Narrow therapeutic index
NTID	Narrow therapeutic index drug
NTRD	Narrow therapeutic range drug
PBBM	Physiologically based biopharmaceutics modeling
PBE	Population bioequivalence
PBPK	Physiologically based pharmacokinetics
PK	Pharmacokinetics
PK–PD	Pharmacokinetics–pharmacodynamics
PMDA	Pharmaceuticals and Medical Devices Agency
PopPK	Population pharmacokinetics
PT-INR	Prothrombin time–international normalized ratio
R	Reference formulation
RSABE	Reference-scaled average bioequivalence
SFDA	Saudi Food and Drug Authority
SHAP	Shapley additive explanations
SWR	Within-subject standard deviation of the reference formulation
SWT	Within-subject standard deviation of the test formulation
T	Test formulation
TDM	Therapeutic drug monitoring
TFDA	Taiwan Food and Drug Administration
VKORC1	Vitamin K epoxide reductase complex subunit 1
WHO	World Health Organization
XGBoost	Extreme gradient boosting
