# Supplementary material for: Narrow Therapeutic Index Drugs: Regulatory Frameworks, Bioequivalence, and Model-Informed Precision Dosing
Source: Pharmaceutics. 2026 Sep 20;18(9):1190. doi: 10.3390/pharmaceutics18091190 (PMC13610058; doi:10.3390/pharmaceutics18091190)
Supplement: Supplementary file 1 [file pharmaceutics-18-01190-s001.zip › pharmaceutics-4528046-supplementary.pdf]

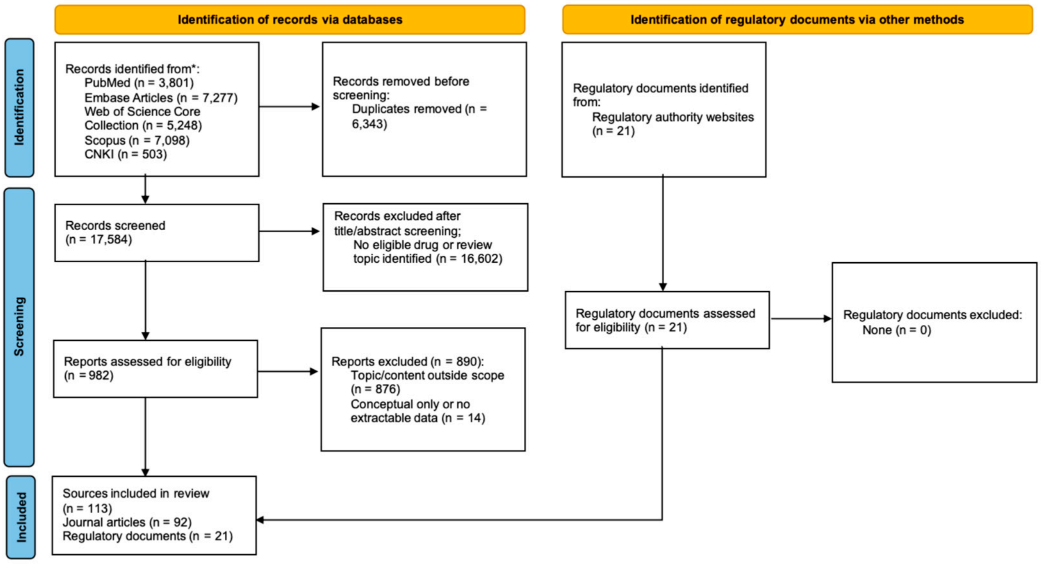


**Figure S1. Literature search flowchart**

## Supplementary Method S1 Database-specific supplementary search strategies

### Search scope and execution

The review's specified publication cutoff was 30 June 2026. Each database query combined three components using OR: NTI AND regulatory/bioequivalence terms; NTI AND modeling/precision-dosing terms; and drug names AND modeling/precision-dosing terms. Synonyms were joined by OR within each concept.

### S1.1 PubMed

Vocabulary and fields: specified MeSH Terms and Title/Abstract terms.
Syntax: OR and AND; quoted terms; suffix asterisk for truncation in the specified free-text terms. No proximity operators were used.
Initial retrieval count: 3,801.

((("narrow therapeutic index"[Title/Abstract] OR "narrow therapeutic indices"[Title/Abstract] OR "narrow therapeutic window"[Title/Abstract] OR "narrow therapeutic windows"[Title/Abstract] OR "narrow therapeutic range"[Title/Abstract] OR "narrow therapeutic ranges"[Title/Abstract] OR "narrow therapeutic ratio"[Title/Abstract] OR "narrow therapeutic ratios"[Title/Abstract] OR "critical dose drug"[Title/Abstract] OR "critical dose drugs"[Title/Abstract]) AND (("Therapeutic Equivalency"[MeSH Terms] OR "Drug Substitution"[MeSH Terms] OR "Drug Approval"[MeSH Terms] OR "bioequivalen*"[Title/Abstract] OR "generic drug"[Title/Abstract] OR "generic drugs"[Title/Abstract] OR "generic product"[Title/Abstract] OR "generic products"[Title/Abstract] OR "substitut*"[Title/Abstract] OR "switch*"[Title/Abstract] OR "interchangeab*"[Title/Abstract] OR "biocreep"[Title/Abstract] OR "reference-scaled average

```
bioequivalence"[Title/Abstract] OR "RSABE"[Title/Abstract] OR "average bioequivalence"[Title/Abstract] OR "individual bioequivalence"[Title/Abstract] OR "population bioequivalence"[Title/Abstract] OR "within-subject variability"[Title/Abstract] OR "within subject variability"[Title/Abstract] OR "regulat*"[Title/Abstract] OR "guid*"[Title/Abstract]) OR ("Machine Learning"[MeSH Terms] OR "population pharmacokinetic*"[Title/Abstract] OR "PopPK"[Title/Abstract] OR "pharmacokinetic-pharmacodynamic"[Title/Abstract] OR "pharmacokinetic pharmacodynamic"[Title/Abstract] OR "PK-PD"[Title/Abstract] OR "PK/PD"[Title/Abstract] OR "model-informed precision dosing"[Title/Abstract] OR "model informed precision dosing"[Title/Abstract] OR "MIPD"[Title/Abstract] OR "Bayesian forecast*"[Title/Abstract] OR "Bayesian dosing"[Title/Abstract] OR "physiologically based pharmacokinetic*"[Title/Abstract] OR "PBPK"[Title/Abstract] OR "physiologically based biopharmaceutics"[Title/Abstract] OR "PBBM"[Title/Abstract] OR "virtual bioequivalence"[Title/Abstract] OR "machine learning"[Title/Abstract] OR "deep learning"[Title/Abstract] OR "reinforcement learning"[Title/Abstract] OR "hybrid model*"[Title/Abstract] OR (("Drug Monitoring"[MeSH Terms] OR "therapeutic drug monitoring"[Title/Abstract] OR TDM[Title/Abstract]) AND (Bayesian[Title/Abstract] OR "model-informed"[Title/Abstract] OR "model informed"[Title/Abstract] OR "precision dosing"[Title/Abstract]))))) OR (("warfarin"[Title/Abstract] OR "digoxin"[Title/Abstract] OR "phenytoin"[Title/Abstract] OR "carbamazepine"[Title/Abstract] OR "valproate"[Title/Abstract] OR "valproic acid"[Title/Abstract] OR "divalproex"[Title/Abstract] OR "tacrolimus"[Title/Abstract] OR "vancomycin"[Title/Abstract] OR "voriconazole"[Title/Abstract]) AND ("Machine Learning"[MeSH Terms] OR "population pharmacokinetic*"[Title/Abstract] OR "PopPK"[Title/Abstract] OR "pharmacokinetic-pharmacodynamic"[Title/Abstract] OR "pharmacokinetic pharmacodynamic"[Title/Abstract] OR "PK-PD"[Title/Abstract] OR "PK/PD"[Title/Abstract] OR "model-informed precision dosing"[Title/Abstract] OR "model informed precision dosing"[Title/Abstract] OR "MIPD"[Title/Abstract] OR "Bayesian forecast*"[Title/Abstract] OR "Bayesian dosing"[Title/Abstract] OR "physiologically based pharmacokinetic*"[Title/Abstract] OR "PBPK"[Title/Abstract] OR "physiologically based biopharmaceutics"[Title/Abstract] OR "PBBM"[Title/Abstract] OR "virtual bioequivalence"[Title/Abstract] OR "machine learning"[Title/Abstract] OR "deep learning"[Title/Abstract] OR "reinforcement learning"[Title/Abstract] OR "hybrid model*"[Title/Abstract] OR (("Drug Monitoring"[MeSH Terms] OR "therapeutic drug monitoring"[Title/Abstract] OR TDM[Title/Abstract]) AND (Bayesian[Title/Abstract] OR "model-informed"[Title/Abstract] OR "model informed"[Title/Abstract] OR "precision dosing"[Title/Abstract]))))) AND ("1900/01/01"[Date - Publication] : "2026/06/30"[Date - Publication])
```

### S1.2 Embase

Vocabulary and fields: /exp for the specified Emtree headings; ti,ab,kw for title, abstract, and author-keyword terms.

Syntax: OR and AND; single-quoted terms; suffix asterisk for the specified free-text terms. No proximity operators were used.
Initial retrieval counts: Articles, 7,277; Clinical trials, 222; total, 7,499.

((('narrow therapeutic index':ti,ab,kw OR 'narrow therapeutic indices':ti,ab,kw OR 'narrow therapeutic window':ti,ab,kw OR 'narrow therapeutic windows':ti,ab,kw OR 'narrow therapeutic range':ti,ab,kw OR 'narrow therapeutic ranges':ti,ab,kw OR 'narrow therapeutic ratio':ti,ab,kw OR 'narrow therapeutic ratios':ti,ab,kw OR 'critical dose drug':ti,ab,kw OR 'critical dose drugs':ti,ab,kw) AND (('bioequivalence'/exp OR 'drug substitution'/exp OR 'generic drug'/exp OR 'drug approval'/exp OR 'bioequivalen*':ti,ab,kw OR 'generic drug':ti,ab,kw OR 'generic drugs':ti,ab,kw OR 'generic product':ti,ab,kw OR 'generic products':ti,ab,kw OR 'substitut*':ti,ab,kw OR 'switch*':ti,ab,kw OR 'interchangeab*':ti,ab,kw OR 'biocreep':ti,ab,kw OR 'reference-scaled average bioequivalence':ti,ab,kw OR 'RSABE':ti,ab,kw OR 'average bioequivalence':ti,ab,kw OR 'individual bioequivalence':ti,ab,kw OR 'population bioequivalence':ti,ab,kw OR 'within-subject variability':ti,ab,kw OR 'within subject variability':ti,ab,kw OR 'regulat*':ti,ab,kw OR 'guid*':ti,ab,kw) OR ('machine learning'/exp OR 'population pharmacokinetic*':ti,ab,kw OR 'PopPK':ti,ab,kw OR 'pharmacokinetic-pharmacodynamic':ti,ab,kw OR 'pharmacokinetic pharmacodynamic':ti,ab,kw OR 'PK-PD':ti,ab,kw OR 'PK/PD':ti,ab,kw OR 'model-informed precision dosing':ti,ab,kw OR 'model informed precision dosing':ti,ab,kw OR 'MIPD':ti,ab,kw OR 'Bayesian forecast*':ti,ab,kw OR 'Bayesian dosing':ti,ab,kw OR 'physiologically based pharmacokinetic*':ti,ab,kw OR 'PBPK':ti,ab,kw OR 'physiologically based biopharmaceutics':ti,ab,kw OR 'PBBM':ti,ab,kw OR 'virtual bioequivalence':ti,ab,kw OR 'machine learning':ti,ab,kw OR 'deep learning':ti,ab,kw OR 'reinforcement learning':ti,ab,kw OR 'hybrid model*':ti,ab,kw OR (('therapeutic drug monitoring'/exp OR 'therapeutic drug monitoring':ti,ab,kw OR 'tdm':ti,ab,kw) AND ('bayesian':ti,ab,kw OR 'model-informed':ti,ab,kw OR 'model informed':ti,ab,kw OR 'precision dosing':ti,ab,kw))))) OR (('warfarin':ti,ab,kw OR 'digoxin':ti,ab,kw OR 'phenytoin':ti,ab,kw OR 'carbamazepine':ti,ab,kw OR 'valproate':ti,ab,kw OR 'valproic acid':ti,ab,kw OR 'divalproex':ti,ab,kw OR 'tacrolimus':ti,ab,kw OR 'vancomycin':ti,ab,kw OR 'voriconazole':ti,ab,kw) AND ('machine learning'/exp OR 'population pharmacokinetic*':ti,ab,kw OR 'PopPK':ti,ab,kw OR 'pharmacokinetic-pharmacodynamic':ti,ab,kw OR 'pharmacokinetic pharmacodynamic':ti,ab,kw OR 'PK-PD':ti,ab,kw OR 'PK/PD':ti,ab,kw OR 'model-informed precision dosing':ti,ab,kw OR 'model informed precision dosing':ti,ab,kw OR 'MIPD':ti,ab,kw OR 'Bayesian forecast*':ti,ab,kw OR 'Bayesian dosing':ti,ab,kw OR 'physiologically based pharmacokinetic*':ti,ab,kw OR 'PBPK':ti,ab,kw OR 'physiologically based biopharmaceutics':ti,ab,kw OR 'PBBM':ti,ab,kw OR 'virtual bioequivalence':ti,ab,kw OR 'machine learning':ti,ab,kw OR 'deep learning':ti,ab,kw OR 'reinforcement learning':ti,ab,kw OR 'hybrid model*':ti,ab,kw OR (('therapeutic drug monitoring'/exp OR 'therapeutic drug monitoring':ti,ab,kw OR 'tdm':ti,ab,kw) AND ('bayesian':ti,ab,kw OR 'model-informed':ti,ab,kw OR 'model informed':ti,ab,kw OR 'precision dosing':ti,ab,kw))))) AND [1900-2026]/py

**S1.3 Web of Science**

Syntax: OR and AND; double-quoted terms; suffix asterisk for the specified free-text terms. No proximity operators were used.
Initial retrieval count: 5,248.

((TS=("narrow therapeutic index" OR "narrow therapeutic indices" OR "narrow therapeutic window" OR "narrow therapeutic windows" OR "narrow therapeutic range" OR "narrow therapeutic ranges" OR "narrow therapeutic ratio" OR "narrow therapeutic ratios" OR "critical dose drug" OR "critical dose drugs") AND (TS=("bioequivalen*" OR "generic drug" OR "generic drugs" OR "generic product" OR "generic products" OR "substitut*" OR "switch*" OR "interchangeab*" OR "biocreep" OR "reference-scaled average bioequivalence" OR "RSABE" OR "average bioequivalence" OR "individual bioequivalence" OR "population bioequivalence" OR "within-subject variability" OR "within subject variability" OR "regulat*" OR "guid*") OR TS=("population pharmacokinetic*" OR "PopPK" OR "pharmacokinetic-pharmacodynamic" OR "pharmacokinetic pharmacodynamic" OR "PK-PD" OR "PK/PD" OR "model-informed precision dosing" OR "model informed precision dosing" OR "MIPD" OR "Bayesian forecast*" OR "Bayesian dosing" OR "physiologically based pharmacokinetic*" OR "PBPK" OR "physiologically based biopharmaceutics" OR "PBBM" OR "virtual bioequivalence" OR "machine learning" OR "deep learning" OR "reinforcement learning" OR "hybrid model*" OR (("therapeutic drug monitoring" OR TDM) AND (Bayesian OR "model-informed" OR "model informed" OR "precision dosing"))))) OR (TS=("warfarin" OR "digoxin" OR "phenytoin" OR "carbamazepine" OR "valproate" OR "valproic acid" OR "divalproex" OR "tacrolimus" OR "vancomycin" OR "voriconazole") AND TS=("population pharmacokinetic*" OR "PopPK" OR "pharmacokinetic-pharmacodynamic" OR "pharmacokinetic pharmacodynamic" OR "PK-PD" OR "PK/PD" OR "model-informed precision dosing" OR "model informed precision dosing" OR "MIPD" OR "Bayesian forecast*" OR "Bayesian dosing" OR "physiologically based pharmacokinetic*" OR "PBPK" OR "physiologically based biopharmaceutics" OR "PBBM" OR "virtual bioequivalence" OR "machine learning" OR "deep learning" OR "reinforcement learning" OR "hybrid model*" OR (("therapeutic drug monitoring" OR TDM) AND (Bayesian OR "model-informed" OR "model informed" OR "precision dosing")))))

**S1.4 Scopus**

Syntax: OR and AND; double-quoted terms; suffix asterisk for the specified free-text terms. No proximity operators were used.
Initial retrieval count: 7,098.

((TITLE-ABS-KEY("narrow therapeutic index" OR "narrow therapeutic indices" OR "narrow therapeutic window" OR "narrow therapeutic windows" OR "narrow therapeutic range" OR "narrow therapeutic ranges" OR "narrow therapeutic ratio" OR "narrow therapeutic ratios" OR "critical dose drug" OR "critical dose drugs") AND (TITLE-ABS-KEY("bioequivalen*"

OR "generic drug" OR "generic drugs" OR "generic product" OR "generic products" OR "substitut*" OR "switch*" OR "interchangeab*" OR "biocreep" OR "reference-scaled average bioequivalence" OR "RSABE" OR "average bioequivalence" OR "individual bioequivalence" OR "population bioequivalence" OR "within-subject variability" OR "within subject variability" OR "regulat*" OR "guid*") OR TITLE-ABS-KEY("population pharmacokinetic*" OR "PopPK" OR "pharmacokinetic-pharmacodynamic" OR "pharmacokinetic pharmacodynamic" OR "PK-PD" OR "PK/PD" OR "model-informed precision dosing" OR "model informed precision dosing" OR "MIPD" OR "Bayesian forecast*" OR "Bayesian dosing" OR "physiologically based pharmacokinetic*" OR "PBPK" OR "physiologically based biopharmaceutics" OR "PBBM" OR "virtual bioequivalence" OR "machine learning" OR "deep learning" OR "reinforcement learning" OR "hybrid model*" OR (("therapeutic drug monitoring" OR TDM) AND (Bayesian OR "model-informed" OR "model informed" OR "precision dosing"))))) OR (TITLE-ABS-KEY("warfarin" OR "digoxin" OR "phenytoin" OR "carbamazepine" OR "valproate" OR "valproic acid" OR "divalproex" OR "tacrolimus" OR "vancomycin" OR "voriconazole") AND TITLE-ABS-KEY("population pharmacokinetic*" OR "PopPK" OR "pharmacokinetic-pharmacodynamic" OR "pharmacokinetic pharmacodynamic" OR "PK-PD" OR "PK/PD" OR "model-informed precision dosing" OR "model informed precision dosing" OR "MIPD" OR "Bayesian forecast*" OR "Bayesian dosing" OR "physiologically based pharmacokinetic*" OR "PBPK" OR "physiologically based biopharmaceutics" OR "PBBM" OR "virtual bioequivalence" OR "machine learning" OR "deep learning" OR "reinforcement learning" OR "hybrid model*" OR (("therapeutic drug monitoring" OR TDM) AND (Bayesian OR "model-informed" OR "model informed" OR "precision dosing"))))) AND PUBYEAR AFT 1899 AND PUBYEAR BEF 2027

**S1.5 CNKI**

Syntax: single-quoted terms; + for OR; * for AND. The asterisk in this expression is a set-combination operator.
Initial retrieval count: 503.

**China National Knowledge Infrastructure (CNKI)**
SU=((('narrow therapeutic index' + 'narrow therapeutic indices' + 'narrow therapeutic window' + 'narrow therapeutic windows' + 'narrow therapeutic range' + 'narrow therapeutic ranges' + 'narrow therapeutic ratio' + 'narrow therapeutic ratios' + 'critical dose drug' + 'critical dose drugs')*(('bioequivalence' + 'generic drug' + 'generic drugs' + 'consistency evaluation' + 'drug substitution' + 'interchangeability' + 'drug switching' + 'biocreep' + 'regulation' + 'regulatory guidance' + 'guideline' + 'RSABE' + 'population bioequivalence' + 'individual bioequivalence') +(('population pharmacokinetics' + 'pharmacokinetic/pharmacodynamic' + 'PK/PD' + 'PK-PD' + 'model-informed precision dosing' + 'MIPD' + 'Bayesian forecasting' + 'Bayesian dosing' + 'physiologically based pharmacokinetic' + 'PBPK' + 'PBBM' + 'physiologically based biopharmaceutics' + 'virtual bioequivalence' + 'machine learning' + 'deep learning' + 'reinforcement learning' + 'hybrid model' + 'PopPK' + 'population pharmacokinetic') +(('therapeutic drug monitoring' + 'TDM')*('Bayesian' + 'model-informed' + 'model-guided' +

'precision dosing'))))) + (('warfarin' + 'digoxin' + 'phenytoin' + 'phenytoin sodium' + 'carbamazepine' + 'valproate' + 'valproic acid' + 'sodium valproate' + 'divalproex' + 'divalproex sodium' + 'tacrolimus' + 'vancomycin' + 'voriconazole')* (('population pharmacokinetics' + 'pharmacokinetic/pharmacodynamic' + 'PK/PD' + 'PK-PD' + 'model-informed precision dosing' + 'MIPD' + 'Bayesian forecasting' + 'Bayesian dosing' + 'physiologically based pharmacokinetic' + 'PBPK' + 'PBBM' + 'physiologically based biopharmaceutics' + 'virtual bioequivalence' + 'machine learning' + 'deep learning' + 'reinforcement learning' + 'hybrid model' + 'PopPK' + 'population pharmacokinetic')+(('therapeutic drug monitoring' + 'TDM')*('Bayesian' + 'model-informed' + 'model-guided' + 'precision dosing')))))
